# *Mir21* modulates inflammation and sensorimotor deficits in cervical myelopathy: data from humans and animal models

**DOI:** 10.1093/braincomms/fcaa234

**Published:** 2021-01-21

**Authors:** Alex M Laliberte, Spyridon K Karadimas, Pia M Vidal, Kajana Satkunendrarajah, Michael G Fehlings

**Affiliations:** Division of Genetics and Development, Krembil Research Institute, University Health Network, Toronto, ON M5T2S8, Canada; Institute of Medical Sciences, University of Toronto, Toronto, ON M5T2S8, Canada; Department of Biology, University of Ottawa, Ottawa, ON K1N6N5, Canada; Division of Genetics and Development, Krembil Research Institute, University Health Network, Toronto, ON M5T2S8, Canada; Institute of Medical Sciences, University of Toronto, Toronto, ON M5T2S8, Canada; Division of Neurosurgery, Department of Surgery, University of Toronto, ON M5T2S8, Canada; Division of Genetics and Development, Krembil Research Institute, University Health Network, Toronto, ON M5T2S8, Canada; Department of Basic Science, Biomedical Science Research Lab, Faculty of Medicine, Universidad Católica de la Santísima Concepción, Concepción, Chile; Division of Genetics and Development, Krembil Research Institute, University Health Network, Toronto, ON M5T2S8, Canada; Department of Neurosurgery, Medical College of Wisconsin, Milwaukee, WI 53226, USA; Clement J. Zablocki Veterans Affairs Medical Center, Milwaukee, WI 53295, USA; Department of Physiology, Medical College of Wisconsin, Milwaukee, WI 53226, USA; Division of Genetics and Development, Krembil Research Institute, University Health Network, Toronto, ON M5T2S8, Canada; Institute of Medical Sciences, University of Toronto, Toronto, ON M5T2S8, Canada; Division of Neurosurgery, Department of Surgery, University of Toronto, ON M5T2S8, Canada

**Keywords:** degenerative cervical myelopathy, microRNA, biomarker, inflammation, spinal cord

## Abstract

Degenerative cervical myelopathy is a common condition resulting from chronic compression of the spinal cord by degenerating structures of the spine. Degenerative cervical myelopathy present a wide range of outcomes, and the biological factors underlying this variability are poorly understood. Previous studies have found elevated MIR21-5p in the sub-acute and chronic neuroinflammatory environment after spinal cord injury. As chronic spinal cord neuroinflammation is a major feature of degenerative cervical myelopathy, we hypothesized that MIR21-5p may be particularly relevant to disease pathobiology, and could serve as a potential biomarker. A prospective cohort study of 69 human degenerative cervical myelopathy patients (36 male:33 female) between the ages of 30 and 78 years was performed to identify the relationship between MIR21-5p expression, symptom severity and treatment outcomes. Results from this study identified a positive correlation between elevated plasma MIR21-5p expression, initial symptom severity and poor treatment outcomes. Subsequent validation of these relationships using a mouse model of degenerative cervical myelopathy identified a similar elevation of MIR21-5p expression at 6 and 12 weeks after onset, corresponding to moderate to severe neurological deficits. To further determine how MIR21-5p affects cervical myelopathy pathobiology, this mouse model was applied to a *Mir21* knockout mouse line. Deletion of the *Mir21* gene preserved locomotor function on rotarod and forced swim tests, but also resulted in increased nociception based on tail flick, Von Frey filament and electrophysiological testing. Critically, *Mir21* knockout mice also had reduced spinal cord inflammation, demonstrated by the reduction of Iba1+ microglia by ∼50% relative to wild-type controls. *In vitro* experiments using primary microglial cultures confirmed that MIR21-5p expression was greatly increased after exposure to lipopolysaccharide (pro-inflammatory), Il4 (anti-inflammatory) and hypoxia. *Mir21* knockout did not appear to alter the ability of microglia to respond to these stimuli, as expression of key pro- and anti-inflammatory response genes was not significantly altered. However, target prediction algorithms identified the IL6/STAT3 pathway as a potential downstream target of MIR21-5p, and subsequent *in vitro* testing found that expression of components of the IL6 receptor complex, *Il6ra* and *Il6st,* were significantly higher in *Mir21* knockout microglia. In aggregate, these data show that *Mir21* plays a role in the progression of motor deficits and neuroinflammatory modulation in degenerative cervical myelopathy. Given this role in neuroinflammation, and its association with poor patient outcomes, MIR21-5p represents a potential therapeutic target and a new marker for prognostication.

## Teaser

Degenerative cervical myelopathy is a relatively common spinal cord condition with variable presentation and a wide range of outcomes. Using both human and mouse data, Laliberte *et al.* demonstrate a link between elevated microRNA-21-5p expression and increased severity, inflammation and poor treatment outcomes in degenerative cervical myelopathy.

## Graphical Abstract

**Graphical Abstract:**
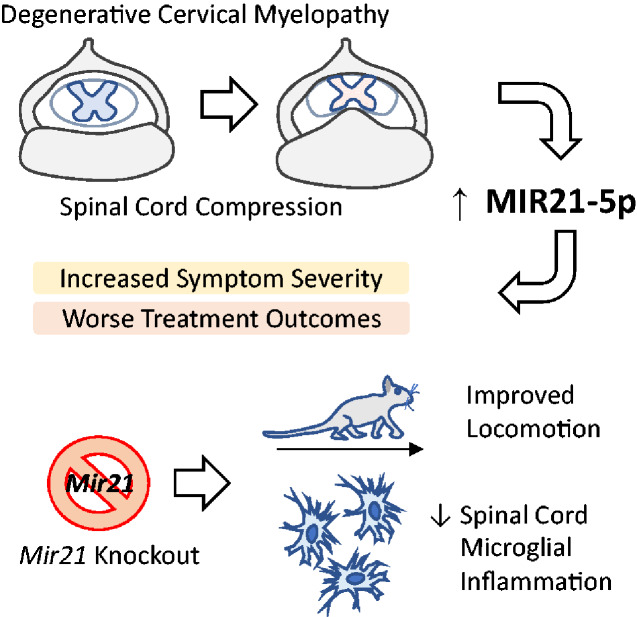

## Introduction

Degenerative cervical myelopathy is a non-traumatic, chronic compressive form of spinal cord injury that is considered to be the most common cause of spinal cord dysfunction worldwide (Nouri *et al.*, 2015). The symptoms of degenerative cervical myelopathy are devastating and highly variable, including broad-based unstable gait, loss of manual dexterity, pain, sensory loss and spasticity (Nurick, 1972, Karadimas *et al.*, 2013a). Surgical decompression of the spinal cord can halt disease progression and can result in substantial neurological recovery, but a subset of degenerative cervical myelopathy patients experiences poor recovery, or even deteriorates further post-operatively (Fehlings *et al.*, 2015, Karadimas *et al.*, 2015b). Unfortunately, the underlying mechanisms that result in this variability in patient outcomes are either unknown or not detectable with existing tools, making accurate prognosis and optimal management of degenerative cervical myelopathy a significant challenge.

Circulating microRNAs have recently emerged as biomarkers for spinal cord injury and numerous other diseases. Also, as regulators of mammalian transcription, disease-related microRNAs have potential as therapeutic targets. To date, there have been no published studies examining microRNA biomarkers in degenerative cervical myelopathy. As such, our goal was to identify a microRNA biomarker with mechanistic links to degenerative cervical myelopathy. Degenerative cervical myelopathy differs from spinal cord injury due to the chronic and progressive nature of spinal cord compression, with chronic ischaemia (Breig *et al.*, 1966, Karadimas *et al.*, 2015b; Vidal *et al.*, 2017) and chronic inflammation (Yu *et al.*, 2011, Karadimas *et al.*, 2015a) being dominant mechanisms. After spinal cord injury, MIR21-5p is one of the few microRNAs to have elevated expression beyond the sub-acute phase (Bhalala *et al.*, 2012), making it a possible mediator of chronic spinal cord inflammation. MIR21-5p is one of the most commonly up-regulated genes in CNS injuries (Liu *et al.*, 2009; Bhalala *et al.*, 2012; Yunta *et al.*, 2012; Karl *et al.*, 2017; Leinders *et al.*, 2017), and its overexpression has been observed in neuroinflammatory (Bergman *et al.*, 2013; Murugaiyan *et al.*, 2015; Harrison *et al.*, 2016) and hypoxic/ischaemic states (Ziu *et al.*, 2011; Cui *et al.*, 2017; Jia *et al.*, 2017).

Given the suspected role of MIR21-5p in hypoxia, inflammation and its overexpression in the chronic phase of spinal cord injury, we hypothesized that MIR21-5p would be induced by the chronic spinal cord compression of degenerative cervical myelopathy. Furthermore, given that MIR21-5p has been implicated in inflammatory gene regulation (Bergman *et al.*, 2013; Wang *et al.*, 2015), we postulated that manipulation of MIR21-5p could significantly reduce the extent of harmful neuroinflammation and neurological deficits observed in degenerative cervical myelopathy animal models. Herein, we demonstrate that MIR21-5p is associated with more severe presentation and worse treatment outcome in a prospective clinical study of patients with degenerative cervical myelopathy. This relationship was further supported by MIR21-5p up-regulation in a degenerative cervical myelopathy mouse model and the observation that deletion of *Mir21* results in significant preservation of motor function. Finally, we also demonstrate that MIR21-5p is up-regulated during microglial activation, and that loss of *Mir21* significantly reduces the number of Iba1+ microglia in the compressed spinal cord. In aggregate, these animal and human patient data implicate MIR21-5p as a critical regulator of neuroinflammation and cervical myelopathy pathology, suggesting potential utility of MIR21-5p as a prognostic biomarker and therapeutic target for cervical myelopathy.

## Materials and methods

### Human plasma microRNA study

**Figure 1:**
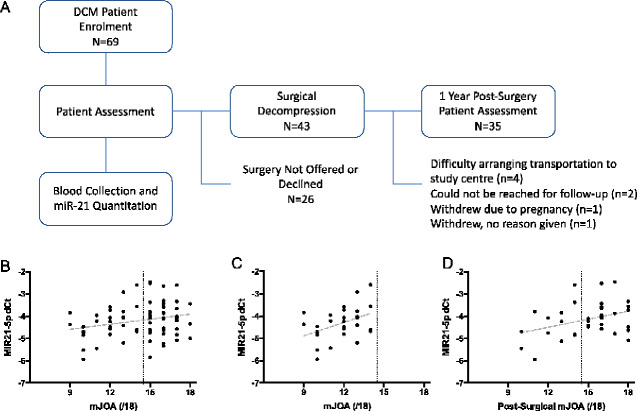
**MIR21-5p expression correlates with severity of neurological deficits in human degenerative cervical myelopathy patients at baseline and after surgical treatment.** (**A**) A schematic representation showing the experimental design and sample size for the degenerative cervical myelopathy (DCM) patient cohort. (**B**) Normalized MIR21-5p expression, denoted as dCt values (where each −1 dCt represents a doubling of expression), was modestly correlated to degenerative cervical myelopathy severity at the time of initial assessment (Pearson’s Correlation test, *r* = 0.248, *P* = 0.049). (**C**) This correlation was stronger in the subset of patients with moderate to severe deficits with an mJOA < 15 (Tetreault *et al.*, 2017) (*r* = 0.424, *P* = 0.025, *n* = 28). (**D**) Initial MIR21-5p expression was also significantly correlated to degenerative cervical myelopathy patient outcome, assessed 1 year after surgical decompression (*r* = 0.368, *P* = 0.030 and *n* = 35).

Study design, data collection and blood analysis protocols were all approved by the University Health Network Research Ethics Board, and consent was obtained from all participants in this study prior to enrolment. The participants were enrolled at the neurosurgical clinic at the Toronto Western Hospital. Inclusion criteria required subjects to be English speakers between 18 and 80 years of age, with imaging confirmation of cervical cord compression and at least one of the following neurological signs: corticospinal motor deficits, atrophy of intrinsic hand muscles, hyper-reflexia, positive Hoffman’s sign, up-going plantar responses, lower-limb spasticity and broad-based unstable gait. Exclusion criteria included: previous spine surgery, symptomatic lumbar stenosis, symptoms due to trauma, uncontrolled or insulin-dependent diabetes, neutropenia, creatinine >1.2 mg/dl, liver enzymes (alanine aminotransferase or aspartate transaminase) three times higher than normal, systemic infection (HIV, hepatitis, etc.), history of hypertension, active malignancy (within the last 5 years), recent history of substance abuse (within last 3 years) and history of myocardial infarction, stroke or heart failure. Initially, 88 degenerative cervical myelopathy patients at the Toronto Western Hospital volunteered with 69 meeting study criteria. These patients were between 30 and 78 years of age, with a mean age of 56.0 ± 10.6 (SD) years at the time of enrolment. Quantitation of MIR21-5p from the 69 degenerative cervical myelopathy patients (36 male:33 female) was performed blinded to patient’s identity or any other clinical data. Severity of degenerative cervical myelopathy was quantified using the modified Japanese Orthopedic Association (mJOA) scale. The mJOA is a multi-faceted assessment tool that grades the impairment caused by upper motor, lower motor, sensory and autonomic dysfunction in degenerative cervical myelopathy patients, providing an overall score out of 18 points (Benzel *et al.*, 1991; Tetreault *et al.*, 2017). Blood was collected after the neurological assessment and prior to any surgical intervention or treatment. Follow-up assessment of patients occurred 1 year after surgery or the initial assessment. Of the 43 patients who underwent surgery, 8 were lost to follow-up (Fig. 1A). Patients lost to follow-up were excluded from analyses examining their outcome.

### Animals

All protocols involving animal use were designed and carried out in accordance with Canadian Council for Animal Care guidelines with ethical approval granted by the institutional animal ethics review board of the University Health Network. All animals were provided standard chow and water *ad libitum* and housed in a barrier containment facility with a 12:12 light/dark cycle. Mouse strains used in this study were commercially available: wild type (WT) (C57bl/6—Jackson Labs# 000664) and *Mir21* knockout (KO) (Jackson Labs# 016856). A total of 55 mice were used, not including those used to derive primary microglial cultures.

### Experimental design

Sample sizes were estimated based on *α* = 0.05 and a minimum power of 0.80 to detect effect sizes observed in comparable experiments from our laboratory. Mice used in this study were female between 2 and 3 months old at the beginning of experiments (*n* = 7/group). Mice of each genotype were allocated to either degenerative cervical myelopathy or sham groups on an alternating basis. Degenerative cervical myelopathy and sham surgeries were performed as described previously (Karadimas *et al.*, 2013b; Vidal *et al.*, 2017). A complete description of the surgery is provided in the Supplementary Material. After surgical induction of degenerative cervical myelopathy, mice were assigned new identifiers by a third party to blind experimenters to animal genotype. Order of behavioural testing was randomized throughout and unblinding occurred at the experimental endpoint.

### Sample collection and tissue processing

At the experimental endpoint, mice were euthanized via isoflurane overdose. For RNA quantification experiments, blood was collected from the left ventricle into EDTA-coated vacutainers (BD Biosciences, Mississauga, Canada) and centrifuged at 1500*g* for 10 min to separate plasma from the cellular pellet. Plasma was then aliquoted and snap frozen in liquid nitrogen prior to storage at −80°C. Mice were transcardially perfused with 1× PBS to clear tissues of blood, and the spinal cords were extracted, frozen on liquid nitrogen and stored at −80°C. For immunohistochemistry, mice were transcardially perfused with 1× PBS, then with ice cold 4% (w/v) paraformaldehyde in 1× PBS. Spinal cords were post-fixed in 4% paraformaldehyde with 10% (w/v) sucrose for 8–16 h at 4°C, followed by a PBS wash and overnight cryoprotection in a 30% (w/v) sucrose in 1× PBS solution. Spinal cords were then embedded in Shandon™ M-1 embedding matrix (ThermoFisher Scientific, Waltham, MA, USA), sliced into 30-μm cross-sections on a cryostat (Leica Microsystems Canada, Richmond Hill) and mounted on Superfrost Plus microscope slides (ThermoFisher Scientific, Waltham, MA, USA).

### RNA extraction

RNA extraction from frozen tissue samples and fresh microglial pellets was performed using the miRCURY cell and plant RNA isolation kit (Exiqon, Vedbaek, Denmark), with supplemental lysis additive for the spinal cord tissue, according to the manufacturer’s instructions. Briefly, 3-mm spinal cord segments were crushed under liquid-nitrogen using a dry ice-cooled mortar and pestle. The recovered powder was then dissolved in lysis solution and passed through a 25-gauge needle to break up remaining tissue and aid in cell lysis prior to beginning the RNA isolation protocol. Eluted RNA quantity and purity were determined using a NanoDrop 1000 spectrophotometer (ThermoFisher Scientific, Waltham, MA, USA) and a BioAnalyzer system (Agilent Technologies, Santa Clara, CA, USA). For the isolation of total RNA from plasma samples, extraction was performed using the miRCURY biofluids RNA kit (Exiqon, Vedbaek, Denmark) according to the manufacturer’s instructions, using 200-μL plasma for human samples and 50-μL plasma (diluted with RNAse-free water to 200 μL) for mouse samples.

### cDNA synthesis

cDNA synthesis for microRNA quantification was performed using the Universal cDNA Synthesis kit II (Exiqon, Vedbaek, Denmark), according to the manufacturer’s instructions. cDNA synthesis for mRNA transcripts was performed using the High-Capacity RNA-to-cDNA™ Kit (ThermoFisher Scientific, Waltham, MA, USA), according to the manufacturer’s instructions. Specific RNA input volumes are listed in Supplementary Table 1. After cDNA synthesis, reactions were cooled to 4°C for immediate use or stored at −20°C.

### Quantitative polymerase chain reaction

Quantitation of MIR21-5p was performed using ExiLENT SYBr^®^-green-based qPCR with locked nucleic acid probes specific to the mature sequence of MIR21-5p (Exiqon, Vedbaek, Denmark). The expression of human plasma MIR21-5p was normalized to MIR423-5p (the most stably expressed micro-RNA from a panel of candidate normalizers—Supplementary Table 2). Relative differences of MIR21-5p between groups were determined using the ΔΔCt method after normalization to endogenous controls, spliceosomal RNA U6 (spinal cord tissue) or MIR16-5p (mouse plasma). For the quantitation of mRNA transcripts (*Tnfa*, *Nos2*, *Arg1*, *Il6*, *Il6ra* and *Il6st*), qPCR was performed using specific Taqman™ probes and Taqman™ Fast Advanced master mix (ThermoFisher Scientific, Waltham, MA, USA). Relative quantities were determined using the ΔΔCt method after normalization to endogenous control (*Gapdh*). All reactions were performed in triplicate and analysed using a 7900HT rtPCR system (ThermoFisher Scientific, Waltham, MA, USA) with the recommended cycling parameters for the qPCR master mix. Specific probe and primer IDs have been included in Supplementary Table 3.

### Rotarod test

Mice were placed on a rotating cylinder suspended at a height that is high enough to induce fall avoidance, but low enough to prevent injury. Prior to data collection, mice were trained at low speeds for a total of 30 min/day for 5 days to gain task-specific competency and increase intra-animal reproducibility. During testing, rotation of the cylinder was gradually increased from 3.5 to 35 rpm. Both the total time spent on the rotarod and the final speed of the mouse were recorded after a fall from the device. Three rotarod runs were collected for each animal and averaged. Sham-operated animals, lacking noticeable locomotor deficits, did not fall from the rotarod after training, and were therefore assigned max values of 35 rpm and 500 s. Since the competency training required for the rotarod test could be considered a form of physical therapy, it was performed only at the experimental endpoint (12 weeks) to avoid potential influence on the progression of neurological deficits in the degenerative cervical myelopathy mouse model.

### Catwalk gait analysis

Analysis of spatiotemporal gait parameters was performed using the Catwalk XT walkway system (Noldus, Leesburg, VA, USA), as described previously (Karadimas *et al.*, 2013b; Vidal *et al.*, 2017). Briefly, mice were recorded while moving across the Catwalk XT walkway. Only uninterrupted runs with a minimum of three continuous step cycles were considered for analysis. Compliant runs were analysed and averaged for each animal.

### Von Frey test

Sensitivity to mechanical stimulus was assessed in mice using Von Frey monofilaments via the frequency method (Bourquin *et al.*, 2006). Mice were allocated to individual 15 × 15 cm chambers set on a 0.3 × 0.3 cm grate (allowing access to the inferior surface of paws from below) and allowed 1 h to acclimatize. Each forepaw and hindpaw was assessed 10 times at each experimental time point by contacting the pad of the paw with a 0.4 g Von Frey hair, producing either a negative (no withdrawal) or positive (withdrawal) response. The total number of positive responses for each paw was tallied and represented as an average value out of 10. Pre-surgical assessment of animals was performed to identify and exclude animals with abnormally high withdrawal responses (>2), resulting in one mouse of each genotype being excluded from pain testing. Mice were given ample time between trials to avoid animal stress and irritation of the target site. Data collection was performed at the same time of day by the same experimenter.

### Tail flick test

Sensitivity to thermal stimulus was assessed in mice using the tail flick test. Mice were held on the Tail Flick Analgesia Meter (IITC Life Sciences, Woodland Hills, LA, USA) with the tail exposed. The heating lamp was initialized for each trial and the time to tail withdrawal was automatically detected. Three trials were performed for each animal at each time point, and the mean value was used for statistical analysis.

### Lumbar dorsal horn local field recordings

Mice were anesthetized using 1% isoflurane and placed prone on a stereotaxic frame to expose the dorsal aspect of the hindlimb. Physiological body temperature was maintained using a heating pad. The sural nerve was dissected in the left hind limb from its origin from the sciatic nerve to the lateral malleolus and the intact sural nerve in the popliteal fossa was electrically stimulated with bipolar silver hook electrodes placed underneath the nerve. The nerve from each animal was covered in mineral oil and stimulated with square-wave pulses of 0.04 ms duration at a frequency of 0.13 ms. The stimulation was initiated first at a minimal current strength required to elicit extracellular potential from the dorsal surface of the lumbar spinal cord (L4) (0.1–0.5 μA). Extracellular potentials were recorded from the dorsal surface of the lumbar spinal cord (L4) with tungsten microelectrodes using Keypoint Portable (Dantec Biomed, Denmark). At a bandwidth of 10–3000 Hz, a total of 100 SEPs were averaged. The experiment was repeated three more times with incremental increases in current strengths by 0.1 μA.

### Forced swim test

Mice were assessed for motor deficits in a sensory-reduced environment using a modified version of the Schnell swim test (Broggini *et al.*, 2016). Individual mice conducted multiple swim trials in a Plexiglas basin containing 25–30°C water and were recorded using a PROMON 501 high-speed camera (AOSTechnologies, Baden, Switzerland) at 50 frames/s. Average swim speed was calculated by measuring the time elapsed to swim a known distance. Only swim trials with continuous swimming (without animals contacting the sides of the basin) were analysed.

### Immunohistochemistry and stereological counting

Microglia and recruited spinal cord macrophages were detected via immunohistochemistry using an anti-Iba1 rabbit polyclonal antibody (1:500, Wako Life Sciences, Mountain View, CA, USA), as described previously (Yu *et al.*, 2011). Fluorescence visualization of Iba1 was enabled via AlexaFluor594-conjugated anti-rabbit IgG (1:400, Sigma-Aldrich, St. Louis, MO, USA) and nuclear fluorescence was provided by 4′,6-diamidino-2-phenylindole (Vector Laboratories, Burlingame, CA, USA). Iba1-positive, 4′,6-diamidino-2-phenylindole-positive cells were estimated throughout the compressed region of the cervical spinal cord using the optical fractionator probe in Stereoinvestigator (MBF Bioscience, Williston, VT, USA).

### Microglial primary cell culture

Mixed glial cultures were prepared from P0–P2-aged pups from C57 or *Mir21* KO breeding pairs as described by others (Vidal *et al.*, 2013; Lam *et al.*, 2017). A more detailed description of the protocol is provided in the Supplementary Material. All fetal bovine serum used for culture experiments was depleted of exosomes by overnight ultracentrifugation at 120 000×*g* using a 70Ti rotor in an Optima L-80 preparative ultracentrifuge (Beckman-Coulter, Brea, CA, USA). To induce pro-inflammatory and anti-inflammatory activation states, 10 ng/ml *Escherichiacoli* lipopolysaccharide (LPS) (Sigma-Aldrich, St. Louis, MO, USA) or 20 ng/ml IL4 (R&D Systems Inc., Minneapolis, MN, USA) was added to the microglial culture, respectively. Hypoxia was induced by culturing microglia for 24 h at 1% O_2_ in a Hypoxia CO_2_ incubator (Nuaire, Plymouth, MA, USA). All microglial treatments were harvested after 24 h, gently washed with 1× PBS, and processed for RNA extraction.

### Statistical analysis

The statistical tests used for all figures and data in this study are listed in Supplementary Table 4. ANOVA assumptions of equality of variance were tested using the Brown–Forsythe test. Data are presented as group means ± SEM unless otherwise noted. All graphs and statistics were created using Prism 6 (GraphPad Software Inc., La Jolla, CA, USA) and SPSS v22 (IBM, Armonk, NY, USA).

### Data availability

The data that support the findings of this study are available from the corresponding author, upon reasonable request

## Results

### MIR21-5p expression in human degenerative cervical myelopathy patient plasma correlates with increased symptom severity and worse treatment outcomes

To examine whether the levels of MIR21-5p were relevant to degenerative cervical myelopathy pathobiology in humans, circulating levels of MIR21-5p were examined in the plasma of 69 patients and compared to the extent of neurological deficits (Fig. 1A). In total, 41 degenerative cervical myelopathy patients (21 male:20 female) with mild symptoms (mJOA ≥ 15) and 28 patients (15 male:13 female) with moderate to severe neurological deficits (mJOA < 15) were examined. Normalized levels of MIR21-5p, denoted as dCt values (where −1 dCt represents a doubling of expression) demonstrated a modest correlation to the mJOA scale (Fig. 1B, Pearson’s correlation test, *r* = 0.238, *P* = 0.049, *n* = 69), the directionality of which indicated that greater expression of MIR21-5p correlates with a greater extent of neurological deficits. This correlation was substantially stronger among individuals with moderate to severe deficits (Fig. 1C, *r* = 0.424, *P* = 0.025, *n* = 28) than in the mild cohort, where no significant correlation was observed (*r* = −0.016, *P* = 0.923, *n* = 41).

To evaluate the potential of MIR21-5p to predict patient outcomes, degenerative cervical myelopathy patients scheduled for decompressive surgery were assessed 1 year after treatment using the mJOA scale. Within this patient cohort, greater pre-operative MIR21-5p expression was correlated with worse patient mJOA scores after surgery (Fig. 1D, *r* = 0.368, *P* = 0.030, *n* = 35), suggesting that elevated levels of MIR21-5p may be detrimental or indicative of poor outcome for human degenerative cervical myelopathy patients undergoing surgical treatment.

### MIR21-5p is up-regulated systemically and in the compressed spinal cords of degenerative cervical myelopathy mice

**Figure 2:**
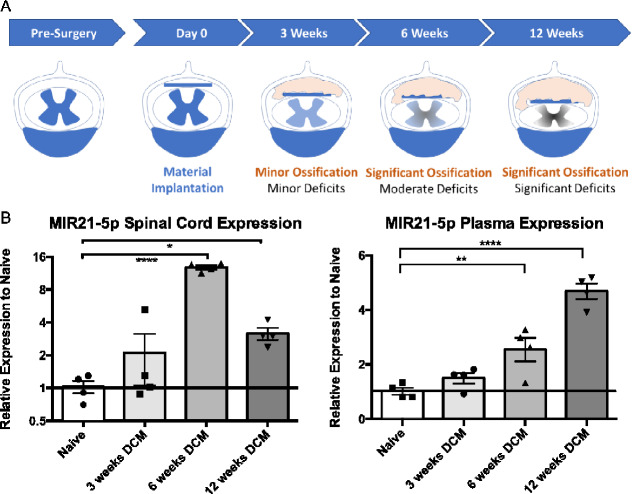
**MIR21-5p expression is increased in a mouse model of degenerative cervical myelopathy.** (**A**) A schematic representation illustrating the timeline of the progressive spinal cord compression induced in the mouse DCM model. (**B**) MIR21-5p expression data from quantitative PCR demonstrate an increase in spinal cord expression at 6 and 12 weeks (ANOVA, Dunnett’s *post-hoc*, *q* = 8.002 and 3.54, respectively, d*f* = 12), and an increase in plasma at 6 and 12 weeks (ANOVA, Dunnett’s *post-hoc*, *q* = 4.272 and 7.453, respectively, d*f* = 12). Spinal cord MIR21-5p was normalized to U6 snRNA, and plasma MIR21-5p to MIR16-5p. *n* = 4 mice. Error bars = ±SEM, *P < 0.05, **P < 0.01 and ****P < 0.0001.

As a means of further examining the observed relationship between MIR21-5p expression and disease severity, expression of MIR21-5p was determined in a mouse model of degenerative cervical myelopathy (Karadimas *et al.*, 2013b; Vidal *et al.*, 2017). This degenerative cervical myelopathy mouse model entails the insertion of a progressively ossifying biomaterial into the spinal canal, causing gradual spinal cord compression. Expression of MIR21-5p was determined 3, 6, and 12 weeks after material implantation and compared to age-matched naïve controls (Fig. 2A) for both plasma and spinal cord samples from the compressed cervical region. As expected, levels of MIR21-5p were elevated in the spinal cords of degenerative cervical myelopathy mice compared to uninjured mice. Mirroring the human data, MIR21-5p expression was greatest in animals with moderate to severe deficits, showing significant up-regulation in the 6- and 12-week cohorts (Fig. 2B, ANOVA, *P* < 0.0001, Dunnett’s *post-hoc*, *q* = 8.002 and 3.54, respectively, d*f* = 12). This increase in MIR21-5p expression at 6 and 12 weeks was also observed systemically in the plasma of degenerative cervical myelopathy mice (Fig. 2B, ANOVA, *P* < 0.0001, Dunnett’s *post-hoc*, *q* = 4.272 and 7.453, respectively, d*f* = 12). As a secondary means of confirming MIR21-5p expression in the compressed spinal cord, *in situ* hybridization for MIR21-5p was also performed on spinal cord tissue sections using a MIR21-5p-specific locked nucleic acid probe. Development of MIR21-5p-probed samples revealed strong precipitate formation in the spinal cord parenchyma of a degenerative cervical myelopathy mouse, but not a *Mir21* KO degenerative cervical myelopathy mouse, demonstrating specificity of the MIR21-5p staining within the degenerative cervical myelopathy spinal cord (Supplementary Fig. 1).

### Deletion of *Mir21* improves locomotor function but with evidence of an antalgic gait pattern

**Figure 3:**
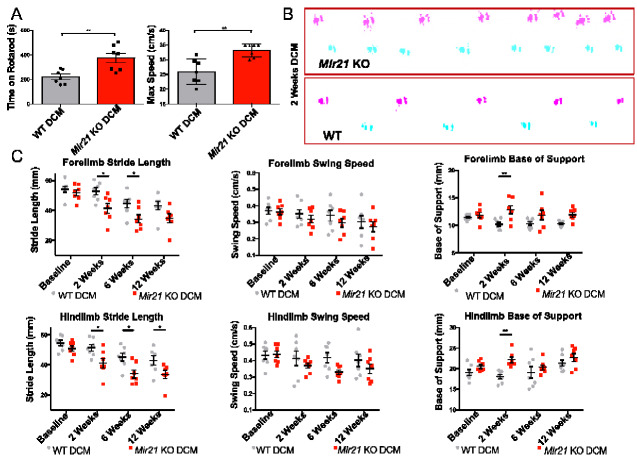
**Deletion of *Mir21* preserves locomotor function on the rotarod test, but generates gait abnormalities during spontaneous movement in mice with degenerative cervical myelopathy.** (**A**) *Mir21* KO animals with DCM for 12 weeks were able to maintain balance for significantly longer than WT degenerative cervical myelopathy animals, and at significantly higher speed while the rotarod accelerated from 3.5 to 35 rpm (*t*-test, *P* = 0.004, *t* = 3.547, and *P* = 0.0017, *t* = 4.031, respectively, d*f* = 12). (**B**) Representative hindlimb traces from 2-week *Mir21* KO and WT degenerative cervical myelopathy mice. *Mir21* KO degenerative cervical myelopathy mice demonstrate a shorter stride length at an early time point that is typically pre-symptomatic in WT animals. (**C**) Repeated measures ANOVA of Catwalk gait results identified significant decreases in forelimb stride length at 2 weeks (Sidak’s *post-hoc*, *t* = 3.083, DF = 48) and 6 weeks (Sidak’s *post-hoc*, *t* = 2.747, DF = 48), and decreases in hindlimb stride length at 2 weeks (Sidak’s *post-hoc*, *t* = 2.846, DF = 48), 6 weeks (Sidak’s *post-hoc*, *t* = 3.144, DF = 48) and 12 weeks (Sidak’s *post-hoc*, *t* = 2.641, DF = 48) in *Mir21* KO degenerative cervical myelopathy mice compared to WT degenerative cervical myelopathy mice. Animal *Mir21* status was identified as a significant factor for hindlimb swing speed (repeated measures ANOVA, *F* = 7.658, DF = 1,12, *P* = 0.017), but multiple comparisons testing did not identify specific differences at any of the time points tested. Forepaw and hindpaw base of support were both significantly increased in *Mir21* KO degenerative cervical myelopathy mice at 2 weeks (Sidak’s *post-hoc*, *t* = 3.722, DF = 48, and *t* = 3.539, DF = 48, respectively). *n* = 7 mice. Error bars = ±SEM, *P < 0.05, **P < 0.01, ***P < 0.001 and ****P < 0.0001.

To determine whether MIR21-5p influences the underlying pathology and the neurological deficits associated with degenerative cervical myelopathy, we examined if the development of locomotor deficits, one of the cardinal symptoms of the human disease, was significantly altered in *Mir21* KO mice. First, we examined the locomotor performance of WT and *Mir21* KO mice using the rotarod test. All sham-operated animals from both WT and *Mir21* KO groups were able to remain on the rotarod at maximum speed and for the entire duration of the test (500 s) after the training period. Among the degenerative cervical myelopathy mice, WT animals, on average, were able to maintain balance on the rotarod for 220.2 ± 21.36 (SEM) s, and had a maximum speed of 25.96 ± 1.587 (SEM) rpm (Fig. 3A). In contrast, *Mir21* KO degenerative cervical myelopathy animals ran ∼70% longer than WT (*P* = 0.004, *t* = 3.547, d*f* = 12), at 376.1 ± 38.40 (SEM) s, and reached an average top speed of 33.21 ± 0.8473 (SEM) rpm, ∼28% faster than WT degenerative cervical myelopathy animals (*P* = 0.0017, *t* = 4.031, d*f* = 12, Fig. 3A).

While rotarod experiments demonstrated significant preservation of locomotor function resulting from *Mir21* deletion, analyses of spontaneous locomotor gait in these animals revealed evidence of gait disruption (Fig. 3B). Specifically, repeated measures ANOVA of Catwalk gait results identified significant decreases in forelimb stride length at 2 weeks (Sidak’s test, *P* = 0.0135, *t* = 3.083 and DF = 48) and 6 weeks (Sidak’s test, *P* = 0.0333, *t* = 2.747 and DF = 48) and decreases in hindlimb stride length at 2 weeks (Sidak’s test, *P* = 0.0257, *t* = 2.846 and DF = 48), 6 weeks (Sidak’s test, *P* = 0.0114, *t* = 3.144 and DF = 48) and 12 weeks (Sidak’s test, *P* = 0.0437, *t* = 2.641 and DF = 48) in *Mir21* KO degenerative cervical myelopathy mice compared to WT degenerative cervical myelopathy mice (Fig. 3C). Animal *Mir21* genotype was identified as a significant factor for hindlimb swing speed (repeated measures ANOVA, *F* = 7.658, DF = 1,12 and *P* = 0.017), but multiple comparisons testing did not identify specific differences at any of the time points tested. Finally, forepaw and hindpaw base of support were both significantly increased in *Mir21* KO degenerative cervical myelopathy mice at 2 weeks (Sidak’s test, *P* = 0.0021, *t* = 3.722, DF = 48 and *P* = 0.0036, *t* = 3.539, DF = 48, respectively). The Catwalk and rotarod data appear contradictory; however, several of the gait parameters identified by the Catwalk system are similarly affected in models of axial or neuropathic pain, common symptoms of degenerative cervical myelopathy (Miyagi *et al.*, 2013; Kameda *et al.*, 2017).

### Sensory assessments demonstrate increased sensitivity to noxious stimuli in *Mir21* knockout mice

**Figure 4:**
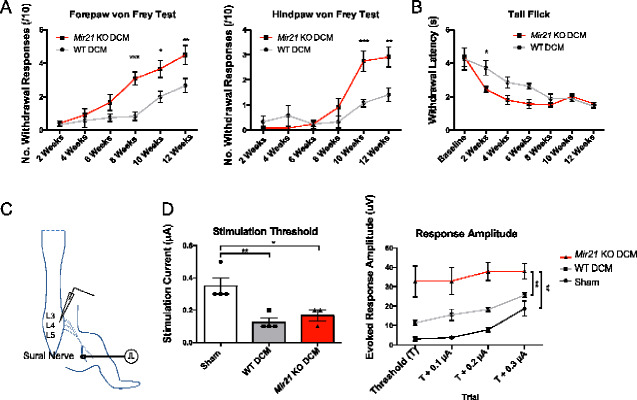
**Sensory assessment using the Von Frey and tail flick tests identify evidence of increased mechanical allodynia and thermal hyperalgesia in *Mir21* KO mice.** (**A**) The frequency of paw withdrawal responses to touch with a calibrated 0.4 g Von Frey monofilament was significantly higher in forepaws of *Mir21* KO **DCM** mice, specifically at 8, 10 and 12 weeks (Repeated Measures ANOVA, Sidak’s *post-hoc*, *t* = 4.212, *t* = 3.12 and *t* = 3.432, respectively, d*f* = 60). Hindpaw Von Frey assessment also found significantly higher withdrawal frequency in *Mir21* KO degenerative cervical myelopathy mice at weeks 10 and 12 (Repeated Measures ANOVA, Sidak’s *post-hoc*, *t* = 4.323 and *t* = 3.891, respectively, DF = 60). (**B**) The latency of withdrawal from a mildly noxious heat stimulus was significantly reduced in *Mir21* KO degenerative cervical myelopathy mice relative to WT degenerative cervical myelopathy at 2 weeks (Repeated Measures ANOVA, Sidak’s *post-hoc*, *t* = 2.862, DF = 70), suggesting increased thermal hyperalgesia in the early stages of disease progression. *n* = 6 mice. (**C**) Schematic representation of dorsal horn field potential recordings during sural nerve stimulation. (**D**) The minimum stimulation threshold and evoked response amplitude from lumbar dorsal horn field recordings. The stimulation threshold from sural nerve stimulation was significantly decreased in both WT degenerative cervical myelopathy and *Mir21* KO degenerative cervical myelopathy mice (ANOVA, Tukey’s *post-hoc*, *P* = 0.0067, *q* = 6.056 and *P* = 0.029, *q* = 4.569, respectively, DF = 8). However, the evoked response amplitude of *Mir21* KO mice (*n* = 3) was significantly higher than both WT degenerative cervical myelopathy (*n* = 4) and Sham (*n* = 4) groups (Repeated Measures ANOVA, Tukey’s *post-hoc*, *P* = 0.008, and *P* = 0.001, respectively, DF = 8). Error bars = ±SEM, *P < 0.05, **P < 0.01, ***P < 0.001 and ****P < 0.0001.

To identify whether loss of *Mir21* altered the manifestation of neuropathic pain in degenerative cervical myelopathy, animals were assessed for mechanical allodynia using the Von Frey test and thermal hyperalgesia using the tail flick test. Using the frequency method, Von Frey filament testing revealed a significant increase in withdrawal frequency in forepaws at 8, 10 and 12 weeks (Fig. 4A, Repeated Measures ANOVA, Sidak’s *post-hoc*, *P* = 0.0005, *t* = 4.212; *P* = 0.0168, *t* = 3.12; and *P* = 0.0065, *t* = 3.432, respectively, d*f* = 60) and in hindpaws at weeks 10 and 12 (Repeated Measures ANOVA, Sidak’s *post-hoc*, *P* = 0.0004, *t* = 4.323; and *P* = 0.0015, *t* = 3.891, respectively, DF = 60). In general, *Mir21* KO degenerative cervical myelopathy animals demonstrated both an earlier onset and a greater overall increase in the frequency of paw withdrawal, indicating a greater magnitude of hypersensitivity than in age-matched WT degenerative cervical myelopathy mice. Results from the tail flick test supported this finding, as the *Mir21* KO degenerative cervical myelopathy mice showed a more rapid withdrawal latency than WT degenerative cervical myelopathy, specifically in the early progression of the disease at the 2-week time point (Fig. 4B, Repeated Measures ANOVA, Sidak’s *post-hoc*, *P* = 0.0382, *t* = 2.862, DF = 70).

While these behavioural responses show that sensory stimulation induces a greater effect in *Mir21* KO degenerative cervical myelopathy animals, both the Von Frey and the tail flick tests rely on a motor response to signal a pain response. As such, differences in motor deficits may confound the nature of the sensory differences between the two genotypes. To distinguish between motor and sensory components of these behavioural tests, we performed *in vivo* electrophysiology in *Mir21* KO and WT mice with degenerative cervical myelopathy or sham surgeries. Stimulation of the sensory aspect of the sural nerve was performed while recording local field potentials from the dorsal horns of the spinal cord at L4. The minimum stimulation threshold to induce dorsal horn field potentials for both *Mir21* KO- and WT degenerative cervical myelopathy mice was reduced relative to sham animals (ANOVA, Tukey’s *post-hoc*, *P* = 0.0067, *q* = 6.056 and *P* = 0.029, *q* = 4.569, respectively, DF = 8), suggesting sensory allodynia, but were not significantly different between WT and *Mir21* KO mice. However, the amplitude of the evoked response in the *Mir21* KO degenerative cervical myelopathy animals was significantly increased relative to WT degenerative cervical myelopathy mice, indicating a greater extent of sensory sensitivity in degenerative cervical myelopathy mice lacking *Mir21* (Repeated Measures ANOVA, Tukey’s *post-hoc*, *P* = 0.008, and *P* = 0.001, respectively, DF = 8).

### *Mir21* KO degenerative cervical myelopathy mice outperform the wild-type cohort in a swimming task that limits mechanosensory feedback

**Figure 5:**
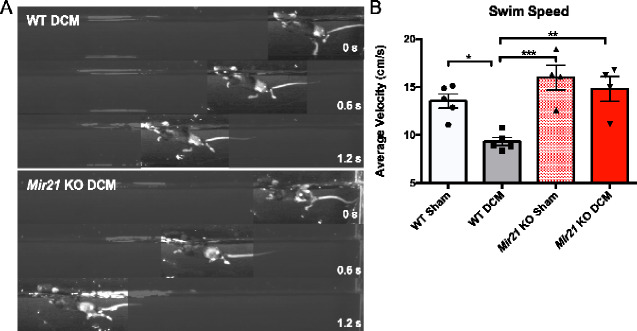
***Mir21* KO mice with degenerative cervical myelopathy swim significantly faster in a forced swim test than WT degenerative cervical myelopathy mice.** (**A**) A representative montage of WT and *Mir21* KO DCM animals swimming over 1.8 s. (**B**) 12-Week *Mir21* KO degenerative cervical myelopathy mice were not significantly different from WT and KO sham animals, but were significantly faster swimmers than WT degenerative cervical myelopathy mice (ANOVA, Tukey’s *post-hoc*, *q* = 5.873, DF = 14). *n* = 5 mice for WT groups, *n* = 4 mice for *Mir21* KO groups. Error bars = ±SEM, *P < 0.05, **P < 0.01, ***P < 0.001 and ****P < 0.0001.

Given that abnormal nociception in *Mir21* KO degenerative cervical myelopathy mice could be confounding their motor output in volitional motor tasks, a swim test was devised as a method to assess locomotor function while minimizing mechanosensory feedback that could be perceived as pain in the degenerative cervical myelopathy mice. This, in theory, would partially isolate the effects of *Mir21* deletion on motor function from those associated with abnormal pain sensation. During the swim test, *Mir21* KO degenerative cervical myelopathy mice swam at an average speed of 14.79 cm/s, a speed that was similar to age-matched sham values (Fig. 5A and B). In contrast, WT degenerative cervical myelopathy mice were significantly slower (9.29 cm/s, Tukey’s test, *P* = 0.0048, *q* = 5.873, d*f* = 14) than *Mir21* KO degenerative cervical myelopathy animals during the swimming task (Fig. 5B), providing support for the observed improvement in motor preservation in *Mir21* KO mice.

### Spinal cord sections show reduced microglial recruitment in *Mir21* KO degenerative cervical myelopathy mice

**Figure 6:**
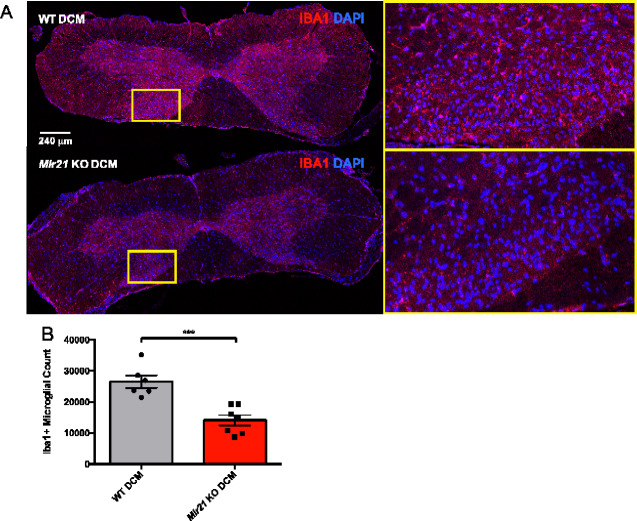
**Iba1 immunohistochemistry and stereological counting identifies a significant reduction of Iba1+ microglia in *Mir21* KO degenerative cervical myelopathy mice relative to WT mice at the 12-week endpoint.** (**A**) Representative images of the compressed regions of WT and *Mir21* KO DCM spinal cords. High-magnification insets highlight the differences in Iba1 staining in the dorsal grey matter. (**B**) A bar graph illustrating the results of stereological counts of Iba1-positive/4′,6-diamidino-2-phenylindole-positive microglia throughout 1.92 mm of the compressed area of the cervical spinal cord (*t*-test, *P* = 0.0006, *t* = 4.785, df = 11). Wild-type degenerative cervical myelopathy (*n* = 6 mice), *Mir21* KO degenerative cervical myelopathy (*n* = 7 mice). Error bars = ±SEM, *P < 0.05, **P < 0.01, ***P < 0.001 and ****(*P* < 0.0001).

Based on the differences in motor and sensory phenotype between WT- and *Mir21* KO degenerative cervical myelopathy mice, we set out to identify differences in spinal cord pathology that might account for these disparities. Since MIR21-5p has been implicated in neuroinflammation (Bergman *et al.*, 2013; Wang *et al.*, 2015), and studies from our laboratory and others have implicated microglia/macrophages as critical mediators of inflammation in degenerative cervical myelopathy models (Yu *et al.*, 2011; Hirai *et al.*, 2013; Moon *et al.*, 2014), we hypothesized that MIR21-5p may affect the microglia/macrophage inflammatory response. Unbiased stereological counting using the Stereoinvestigator Optical Fractionator probe was employed to compare the number of Iba1+ microglial cells in the compressed regions of *Mir21* KO- and WT degenerative cervical myelopathy spinal cords (Fig. 6A). This experiment found that the number of Iba1+ microglia throughout the compressed region of the spinal cord was substantially decreased in *Mir21* KO mice, with microglial counts totalling <50% of the WT degenerative cervical myelopathy values (Fig. 6B, *t*-test, *P* = 0.0006, *t* = 4.785, d*f* = 11).

### MIR21-5p is induced in microglial cells during activation and hypoxia

**Figure 7:**
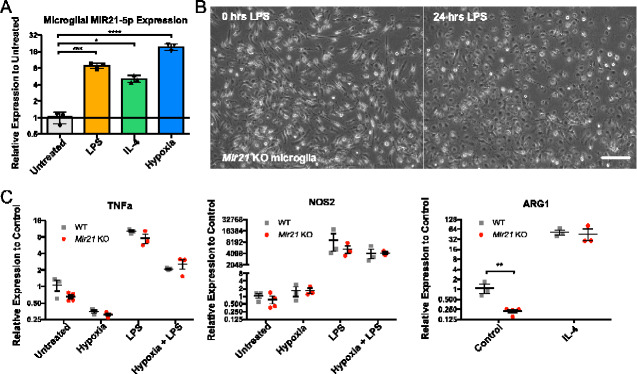
**Primary microglia express MIR21-5p following pro- and anti-inflammatory treatments.** (**A**) MIR21-5p expression was significantly increased relative to controls 24 h after treatment with either 10 ng/ml LPS (ANOVA, Dunnett’s *post-hoc*, *P* = 0.0006, *q* = 6.293, DF = 8), 20 ng/ml IL4 (ANOVA, Dunnett’s *post-hoc*, *P* = 0.0289, *q* = 3.253, DF = 8) or 24-h incubation at 1% O_2_ (ANOVA, Dunnett’s *post-hoc*, *P* < 0.0001, *q* = 14.73, DF = 8). Untreated microglial cultures (*n* = 3), LPS-treated (*n* = 3), IL4-treated (*n* = 3) and Hypoxia-treated (*n* = 3). (**B**) Representative image showing *Mir21* KO microglia retracting processes and adopting the classical amoeboid morphology by 12 h after the addition of LPS (scale bar = 160 μm). (**C**) Typical markers of M1- and M2-polarized macrophages are not differentially expressed between WT and *Mir21* KO (*Mir21* KO) microglia after the addition of pro- and anti-inflammatory stimuli. *Mir21* genotype was not a significant factor for *Tnfa* (two-way ANOVA, *P* = 0.0879, *F* = 3.279, D*F*_(n, d)_ = 1,17) or *Nos2* (two-way ANOVA, *P* = 0.2920, *F* = 1.182, DF_(n, d)_ = 1,17). There was a statistically significant effect of genotype on *Arg1* (two-way ANOVA, *P* = 0.0046, *F* = 13.98, DF_(n, d)_ = 1,9), but multiple comparisons testing found this difference only for untreated microglia (Sidak’s *post-hoc*, *P* = 0.0020, *t* = 4.787, DF = 9), and not those treated with IL4 (Sidak’s *post-hoc*, *P* = 0.7862, *t* = 0.6408, DF = 9). WT untreated microglia (*n* = 3), WT hypoxia (*n* = 3), WT LPS (*n* = 3), WT LPS + hypoxia (*n* = 3), WT IL4 (*n* = 3), *Mir21* KO untreated (*n* = 4), *Mir21* KO hypoxia (*n* = 3), *Mir21* KO LPS (*n* = 3), *Mir21* KO LPS + hypoxia (*n* = 3), *Mir21* KO IL4 (*n* = 3). Error bars = ±SEM, *P < 0.05, **P < 0.01, ***P < 0.001 and ****P < 0.0001.

The reduction in recruited microglia in *Mir21* KO mice suggested that MIR21-5p potentially plays a role in the inflammatory activation of microglia in degenerative cervical myelopathy. To determine whether MIR21-5p is related to the activation of microglia, we isolated primary microglia from the cortex of P0-P2 mice and treated them with either LPS to induce a classical pro-inflammatory phenotype, IL4 to induce an M2 alternative activation state or hypoxia (1% O_2_) for 24 h. All cell conditions demonstrated a significant increase in MIR21-5p relative to control, with the greatest increases resulting from LPS (ANOVA, Dunnett’s *post-hoc*, *P* = 0.0006, *q* = 6.293, DF = 8) and hypoxia treatments (ANOVA, Dunnett’s *post-hoc*, *P* < 0.0001, *q* = 14.73, DF = 8), with relative increases of ∼8- and 20-fold, respectively (Fig. 7A).

### *Mir21* knockout does not impair microglial activation or polarization

To determine whether MIR21-5p is necessary for microglial polarization or viability, primary microglia were also isolated from P0-P2 *Mir21* KO pups. Microglial yield from *Mir21* KO was similar to WT, with three P2 pups yielding between 4–5 × 10^5^ microglial cells in all primary microglial isolations, and thus suggesting that viability was not substantially affected by *Mir21* deletion. Characterization of the microglia did not reveal any obvious abnormalities in cell morphology, and additions of pro-inflammatory treatments produced the expected morphological changes (Fig. 7B), specifically the retraction of processes and the adoption of an amoeboid migratory mode. With respect to the inflammatory phenotype of the microglia, expression of M1 macrophage polarization markers including *Nos2* and *Tnfα* was not significantly different between WT and *Mir21* KO microglia in either the resting state or the following induction with LPS (Fig. 7C). Alternative activation (M2) marker *Arg1* was moderately lower in the untreated *Mir21* KO microglia (two-way ANOVA, Sidak’s *post-hoc*, *P* = 0.002, *t* = 4.787, DF = 9), but induction of the M2 phenotype with IL4 did not result in significant differences in *Arg1* between the two genotypes (Fig. 7C). As such, it appeared as though the loss of *Mir21* did not significantly impact the ability of the microglia to respond to pro- and anti-inflammatory cues.

### *Mir21* knockout alters IL6 signal transducer expression during microglial inflammatory challenge

**Figure 8:**
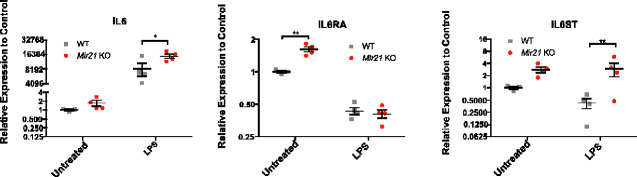
**Deletion of *Mir21* results in significantly higher expression of *Il6*, *Il6ra* and *Il6st* in primary microglia.** *Mir21* genotype was a statistically significant factor for *Il6* (two-way ANOVA, *P* = 0.0112, *F* = 9.263, DF_(n, d)_ = 1,11), *Il6ra* (two-way ANOVA, *P* = 0.0190, *F* = 7.538, DF_(n, d)_ = 1,11) and *Il6st* (two-way ANOVA, *P* = 0.0034, *F* = 13.83, DF_(n, d)_ = 1,11). Specifically, expression of *Il6* and *Il6st* was significantly increased in *Mir21* KO microglia compared to WT microglia after the addition of 10 ng/ml LPS (Sidak’s *post-hoc*, *P* = 0.0469, *t* = 2.623; *P* = 0.0095, *t* = 3.257, respectively, DF = 11). *Il6ra* was not different between genotypes after the addition of LPS, but was higher in *Mir21* KO microglia in the untreated group (Sidak’s *post-hoc*, *P* = 0.0023, *t* = 4.357, DF = 11). WT untreated microglial cultures (*n* = 3), WT LPS (*n* = 4), *Mir21* KO untreated (*n* = 4), *Mir21* KO LPS (*n* = 4). Error bars = ±SEM, *P < 0.05, **P < 0.01, ***P < 0.001 and ****P < 0.0001.

To determine how the loss of MIR21-5p might affect microglial function, we performed a predictive target analysis using a number of target prediction algorithms (TargetScan, RNA22 and miRdB) and a validated target database (TarBase). Aggregation of the results of these analyses revealed a number of predicted targets for MIR21-5p in the IL6/STAT3 pathway, a significant signalling pathway involved in both inflammation (Yasukawa *et al.*, 2003; Fernando *et al.*, 2014) and neuropathic pain (Dominguez *et al.*, 2008; Hori *et al.*, 2016; Molet *et al.*, 2016) (Supplementary Table 5). We therefore examined the mRNA expression of three of the predicted targets (*Il6*, *Il6ra*, and *Il6st*) in both the quiescent and the LPS-activated states of WT and *Mir21* KO microglia. For the predicted targets, *Il6*, *Il6ra* and *Il6st*, *Mir21* genotype was a significant factor in the analysis (two-way ANOVA, *P* = 0.0112, *P* = 0.0190, *P* = 0.0034, respectively). Multiple comparison testing identified *Il6* and *Il6st* as significantly higher in LPS-treated *Mir21* KO microglia (Fig. 8, Sidak’s *post-hoc*, *P* = 0.0469, *t* = 2.623; *P* = 0.0095, *t* = 3.257, respectively, DF = 11), whereas *Il6ra* was higher in the untreated *Mir21* KO microglial cultures (Sidak’s *post-hoc*, *P* = 0.0023, *t* = 4.357, DF = 11).

## Discussion

Due to the gradual and variable nature of degenerative cervical myelopathy development, the molecular determinants of degenerative cervical myelopathy pathobiology have largely remained elusive, yielding few molecular biomarkers or therapeutic targets. Using the combination of prospective human data, a degenerative cervical myelopathy animal model, and *Mir21* KO mice, this study demonstrated a link between MIR21-5p, symptom severity and progression, and microglial inflammation in the compressed spinal cord. Given the known relevance of microglial inflammation to degenerative cervical myelopathy pathobiology (Yu *et al.*, 2011; Hirai *et al.*, 2013; Takeura *et al.*, 2019), we posited that MIR21-5p’s relationship to degenerative cervical myelopathy motor deficits could be mediated through microglia. Subsequent *in vitro* experiments confirmed the induction of MIR21-5p during microglial activation, and identified the IL6/STAT3 pathway as a potential target for MIR21-5p’s effect on microglia. While further research will likely be required to determine the relevance of the proposed MIR21-5p/IL6/STAT3 molecular pathway to degenerative cervical myelopathy pathobiology, this study demonstrates the importance of MIR21-5p in disease progression and suggests a plausible microglial-dependent mechanism for this effect.

The precise role of MIR21-5p in inflammation remains an area of controversy, as several studies have identified its expression under pro- or anti-inflammatory conditions (reviewed in Gaudet *et al.*, 2018). Interestingly, IL6 signalling has a similarly complex and context-dependent role in pro-inflammatory (Romano *et al.*, 1997; McLoughlin *et al.*, 2005; Fielding *et al.*, 2008) and anti-inflammatory mechanisms. Within macrophages, IL6/STAT3 signalling has been associated with the enhancement of M2 anti-inflammatory polarization (Yasukawa *et al.*, 2003; Fernando *et al.*, 2014). Manipulation of MIR21-5p expression in macrophages is consistent with antagonism of IL6/STAT3, as MIR21-5p expression inhibits M2 anti-inflammatory polarization, and *Mir21* KO rescues M2 polarization and increases STAT3 activation (Wang *et al.*, 2015), suggesting a pro-inflammatory role of MIR21-5p in macrophages. Consistent with that conclusion, our results demonstrate a reduction of Iba1+ microglia/macrophages in *Mir21* KO degenerative cervical myelopathy mice.

Despite the noted decrease in Iba1+ microglia, *Mir21* KO mice experienced a greater degree of mechanical and thermal hypersensitivity than WT degenerative cervical myelopathy animals. A potential effect of MIR21-5p on nociception was anticipated, as some recent studies have identified increased levels of MIR21-5p in neuropathic pain states (Hori *et al.*, 2016; Karl *et al.*, 2017; Leinders *et al.*, 2017; Zhong *et al.*, 2019). However, microglia have well-established roles in the genesis of neuropathic pain, and a reduction in local microglia accompanied by increased pain sensitivity was unexpected. While further research will be required to determine the precise mechanism for this increase in pain sensitivity, the proposed role of MIR21-5p in the regulation of the IL6/STAT3 pathway could potentially influence this phenomenon. Intrathecal administration of IL6 is sufficient to generate mechanical allodynia (DeLeo *et al.*, 1996), and it has previously been demonstrated that activation of both microglial and astrocyte STAT3 is central to neuropathic pain (Dominguez *et al.*, 2008, 2010; Tsuda *et al.*, 2011; Liu *et al.*, 2013). Microglial STAT3 activation occurs after rat peripheral nerve injury (Dominguez *et al.*, 2008), and IL6 KO mice do not develop neuropathic pain, nor the expected increase of MIR21-5p after injury (Hori *et al.*, 2016), suggesting a possible reciprocal relationship between MIR21-5p and IL6/STAT3 signalling. In line with these studies and the proposed inhibitory relationship of MIR21-5p on IL6/STAT3 signalling, our results support an antagonistic role of MIR21-5p in the development of neuropathic pain.

Given that MIR21-5p appears related to the underlying pro-inflammatory signalling in degenerative cervical myelopathy, MIR21-5p could represent a useful biomarker for the clinical population. The extent of compression determined from spinal cord imaging is an insufficient predictor of future neurological deficits, particularly in mild or non-myelopathic cases (Oshima *et al.*, 2012; Li *et al.*, 2014; Kovalova *et al.*, 2016). While MIR21-5p expression in the human cohort was more strongly correlated to severe cases, it is possible that outliers with high MIR21-5p expression in the mild patient group may be those with greater risk of imminent deterioration. This information would be extremely valuable for clinical decision making and the prioritization of surgical intervention, but additional longitudinal studies of early-stage/mild degenerative cervical myelopathy patients will be required to evaluate MIR21-5p’s utility for this application. However, based on the existing data, it appears that high MIR21-5p expression is related to worse patient outcomes after surgical intervention. This prognostic information may provide the incentive to develop complementary treatment strategies for individuals with lower potential for recovery. Further validation of MIR21-5p’s relationship to poor surgical outcomes, and determination of its responsiveness to anti-inflammatory treatment, would be valuable in determining its potential utility as a degenerative cervical myelopathy clinical biomarker.

While this study underlines a previously unknown role of MIR21-5p in degenerative cervical myelopathy pathobiology, there are some caveats that limit the interpretation of our results. One limitation of the *in vivo* mouse experiments was that all of the mice were female, limiting generalizability of the results. However, no differences in MIR21-5p expression were observed in human samples between sexes (Supplementary Fig. 2, *P* = 0.2148, *t* = 1.252, d*f* = 67), and hence sex differences in the *Mir21* KO degenerative cervical myelopathy phenotype would not necessarily be expected. Furthermore, to constrain the scope of this research, we primarily focussed on the mechanism of MIR21-5p in microglia/macrophages. However, MIR21-5p has also been implicated in the function of other cells types in neurological injury models. In spinal cord injury, MIR21-5p is reported to play a role in the activation of reactive astrocytes, astrocyte hypertrophy and glial scar formation (Bhalala *et al.*, 2012). Other studies have also reported anti-apoptotic functions of MIR21-5p in neurons after spinal cord (Hu *et al.*, 2013) and brain injury (Ge *et al.*, 2014), whereas another has implicated MIR21-5p binding to TLR-7 as a potential mediator of pro-inflammatory neuron apoptosis (Yelamanchili *et al.*, 2015). Given the diverse mechanisms described, we acknowledge that MIR21-5p potentially has multiple roles depending on the pathological context, and therefore recommend a relatively narrow interpretation of these results in the context of non-traumatic, compressive CNS injury. Also, while our data indicate a regulatory role for MIR21-5p in the IL6 signalling pathway, this study does not directly examine the downstream consequences of *Il6*, *Il6ra*, and *Il6st* de-repression, nor does it exclude the involvement of other signalling pathways relevant to microglial function. Future investigations will be required to further examine the proposed link between MIR21-5p/IL6/STAT3 interactions and microglial function in degenerative cervical myelopathy.

## Conclusion

In conclusion, the results from both human and animal experiments suggest that MIR21-5p is implicated in the development of neurological deficits in degenerative cervical myelopathy, likely via a pro-inflammatory mechanism. This observed relationship between MIR21-5p expression and patient outcomes should provide useful prognostic information and mechanistic insights for future studies of degenerative cervical myelopathy and justifies further examination of MIR21-5p as a potential biomarker of disease progression.

## Supplementary material

Supplementary material is available at *Brain Communications* online.

## Supplementary Material

fcaa234_Supplementary_Data

## Abbreviations

DCM =: degenerative cervical myelopathy
KO =: knockout
LPS =: lipopolysaccharide
mJOA =: modified Japanese Orthopedic Association
WT =: wild type
