# Supplementary material for: Mir21 modulates inflammation and sensorimotor deficits in cervical myelopathy: data from humans and animal models
Source: Brain Commun. 2021 Jan 21;3(1):fcaa234. doi: 10.1093/braincomms/fcaa234 (PMC7878254; doi:10.1093/braincomms/fcaa234)
Supplement: fcaa234_Supplementary_Data [file fcaa234_supplementary_data.zip › Supplementary_material.pdf]

Supplementary Materials:

Supplementary Materials and Methods

Degenerative Cervical Myelopathy and Sham Surgical Protocols

Briefly, animals were pretreated with prophylactic antibiotics (Clavamox, PO, ~14 mg/kg) and provided peri-operative analgesia (buprenorphine, SC, 2x daily, 0.05mg/kg) and antibiotics for the first 3 days after surgery. The dorsal skin and superficial muscle layers were dissected to expose the dorsal aspect of the cervical spinal column in isoflurane anaesthetized mice. Ligamentous structures attached to the C4-C7 laminae were carefully removed, and the inferior surface of the laminae was scratched to disrupt the periosteum. In degenerative cervical myelopathy mice, a folded sheet of polyaromatic ether was carefully inserted between the spinal cord and dorsal laminae spanning the C5-C6 segments(Karadimas *et al.*, 2013*b*). For sham animals, all procedures were kept consistent, with the exception that the polyaromatic ether was inserted, then removed to simulate and control for any potential minor trauma associated with insertion. Finally, to check that the spinal cord and spinal roots were not acutely damaged from the procedure, all mice were examined to 24 hours after surgery for signs of neurological deficits. Any indication of motor deficit resulted in the exclusion of the animal from further study.

In Situ Hybridization

In situ hybridization for MIR21-5p and a positive control, U6 spliceosomal RNA, was performed as previously described(Obernosterer *et al.*, 2007). All solutions for in situ hybridization were ordered RNAse-free or prepared overnight with 0.1% Diethylpyrocarbonate (DEPC, Sigma-Aldrich, St Louis, USA) and autoclaved. Briefly, spinal cord cryosections were

dried, post-fixed with 4%PFA, and washed in 1X PBS. Samples were then submerged in an RNAse-free solution containing triethanolamine, hydrochloric acid and acetic anhydride to acetylate positively charged amino groups and reduce background. Following washing in 1X PBS, tissue sections were permeabilized through treatment with 5 µg/mL RNA grade proteinase K (ThermoFisher Scientific, Waltham, USA) for 5 minutes, and subsequently washed in 1X PBS. Slides were transferred to a humidified hybridization chamber and hybridization solution (50% formamide, 5X SSC buffer, 5X Denhardt's buffer, 500 µg/mL Salmon Sperm DNA, 200 µg/mL Yeast tRNA, 0.02 g/mL blocking reagent (Roche, Basel, Switzerland)) was applied to the tissue before incubation for 4 hours at room temperature. Specific digoxigenin-labeled LNA probes were added to hybridization solution containing 0.25% CHAPS and 0.5% Tween-20, and heat-denatured for 5 minutes at 80˚C before cooling on ice, applying probes to the tissue sections and covering them with Lifterslips™ (ThermoFisher Scientific, Waltham, USA). Slides were then incubated overnight at 53 ˚C to allow probe hybridization. After hybridization, slides were washed in 0.2X Saline-Sodium Citrate and Tris-buffered saline prior to immunohistochemistry using alkaline phosphatase-conjugated anti-digoxigenin sheep polyclonal antibodies (1:500, Roche, Basel, Switzerland). Alkaline phosphatase activity was visualized using the chromogenic nitro-blue tetrazolium and 5-bromo-4-chloro-3'-indolyphosphate substrate (Vector Laboratories, Burlingame, USA).

Primary Microglia Isolation

The cerebellum and meninges were removed from the brain and the remaining brain tissue was minced and dissociated via trituration in cold DMEM. The dissociated cells were then filtered using 40 µm strainers (ThermoFisher Scientific, Waltham, USA), and centrifuged at 300 x g for

10 minutes. Cell pellets were resuspended in DMEM + 10% exosome-depleted fetal bovine serum (FBS, Wisent Bio-Products, St-Bruno, Canada) and seeded in T75 flasks. After 5 days, microglia were isolated from the mixed glial culture by shaking the flasks for 4 hours on an orbital shaker at approximately 70 rpm. The microglia-containing supernatant was collected from the flasks and seeded onto 25mm glass coverslips at a density of 1-2 $x10^5$.

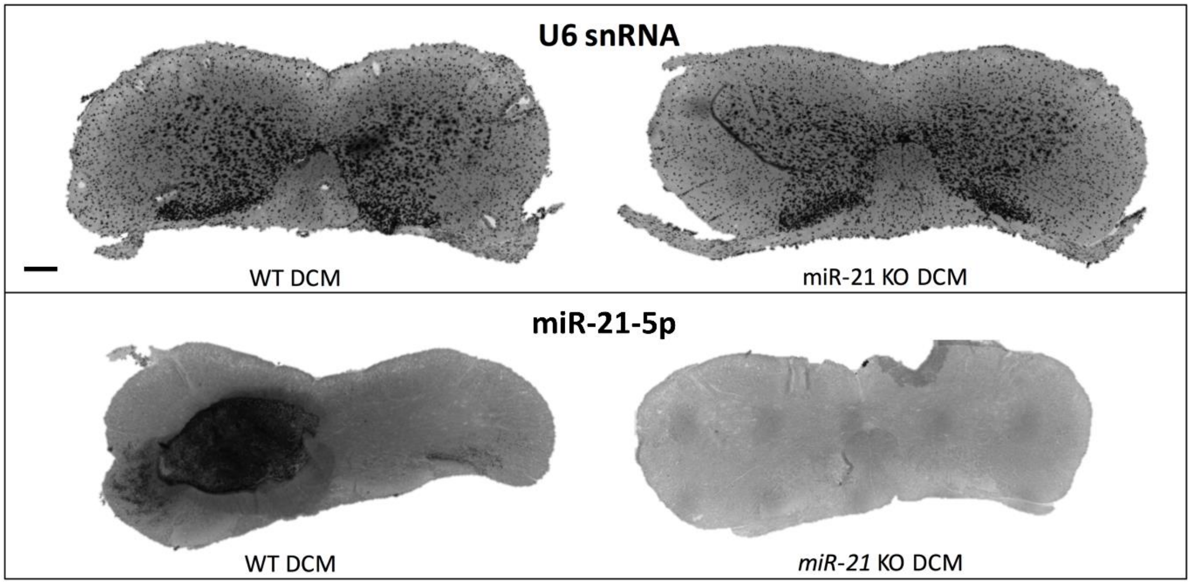

Supplementary Figure 1**. In situ hybridization demonstrates localized overexpression of MIR21-5p in wild type degenerative cervical myelopathy spinal cords.** Chromogenic staining of MIR21-5p and positive control U6 snRNA was performed using in situ hybridization with digoxigenin-labeled locked nucleic acid probes, and subsequently visualized using alkaline phosphatase-conjugated anti-digoxigenin antibodies and the nitro-blue tetrazolium chloride/5-bromo-4-chloro-3'-indolyphosphate p-toluidine salt substrate system. Spinal cord sections from *Mir21* knockout degenerative cervical myelopathy (**DCM**) mice were used as negative controls to demonstrate the hybrization specificity. Scale bar = 240 μm.

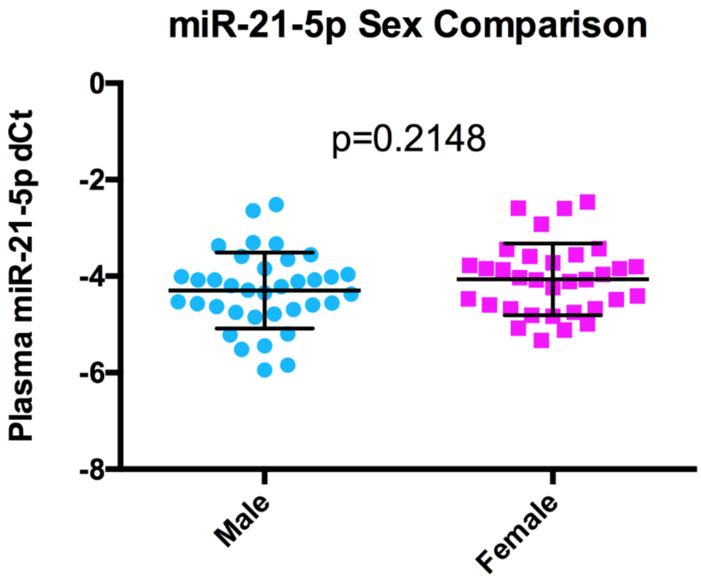

Supplementary Figure 2. **A comparison of plasma MIR21-5p expression between male and female human degenerative cervical myelopathy subjects.** No significant difference was observed between male (n=36) and female (n=33) subjects (t-test, p=.2148, t=1.252, df=69). Error bars = ± SD.

Supplementary Table 1. Details of RNA extraction and cDNA synthesis methodology for the various sample types used in this study.

**Sample Type**	**qPCR Target**	**RNA Extraction Kit**	**cDNA Synthesis Kit**	**RNA Input for cDNA Synthesis**
Human plasma	microRNA	miRCURY Biofluids RNA Isolation Kit	Universal cDNA Synthesis kit II	RNA eluate from 16 µL plasma
Mouse plasma	microRNA	miRCURY Biofluids RNA Isolation Kit	Universal cDNA Synthesis kit II	RNA eluate from 4 µL plasma
Mouse spinal cord	microRNA	miRCURY Cell and Plant RNA Isolation Kit*<br>*with lysis additive	Universal cDNA Synthesis kit II	20 ng / 20 µL reaction
Mouse spinal cord	mRNA	miRCURY Cell and Plant RNA Isolation Kit*<br>*with lysis additive	High-Capacity RNA-to-cDNA™ Kit	100 ng/ 20 µL reaction
Microglial culture	microRNA	miRCURY Cell and Plant RNA Isolation Kit	Universal cDNA Synthesis kit II	20 ng / 20 µL reaction
Microglial culture	mRNA	miRCURY Cell and Plant RNA Isolation Kit	High-Capacity RNA-to-cDNA™ Kit	100 ng/ 20 µL reaction

Supplementary Table 2. Inter-subject variation of candidate microRNAs frequently used for normalization in plasma expression studies. Values were obtained from 10 healthy control, 20 mild, and 10 moderate to severe degenerative cervical myelopathy subjects (n=40).

Candidate Normalization Gene	Mean Ct	Standard Deviation
**MIR423-5p**	**29.31**	**± 0.65**
MIR93-5p	26.08	± 0.69
MIR425-5p	28.40	± 0.74
MIR103a-3p	26.26	± 0.97
MIR191-5p	27.94	± 1.20

Supplementary Table 3. A list of the probes and primers used for qPCR assays.

Gene ID	Supplier	Assay Number	Fluorescent Tag
Il6	Thermofisher Scientific	Mm00446190_m1	FAM
Il6ra	Thermofisher Scientific	Mm01211445_m1	FAM
Il6st	Thermofisher Scientific	Mm00439665_m1	FAM
Tnfa	Thermofisher Scientific	Mm00443258_m1	FAM
Arg1	Thermofisher Scientific	Mm00475988_m1	FAM
Nos2	Thermofisher Scientific	Mm00440502_m1	FAM
Gapdh	Thermofisher Scientific	Mm99999915_g1	FAM
U6 snRNA	Exiqon (Acquired by Qiagen)	YP00203907	N/A (SyBr qPCR)
MIR16-5p	Exiqon (Acquired by Qiagen)	YP00205702	N/A (SyBr qPCR)
MIR423-5p	Exiqon (Acquired by Qiagen)	YP00205624	N/A (SyBr qPCR)
MIR21-5p	Exiqon (Acquired by Qiagen)	YP00204230	N/A (SyBr qPCR)

Supplementary Table 4. Statistical tests used for all figures and data in this study.

**Statistical Test**	**Figures / Data**
Pearson Correlation test	Fig. 1 B-C
One-way ANOVA with Dunnett's post-hoc test	Figs. 2B and 7A
Independent samples t-test	Figs. 3A, 6B
Repeated measures ANOVA with Sidak's post hoc test	Figs. 3C, 4A, and 4B
One-way ANOVA with Tukey's post hoc test	Fig. 5B
Two-way ANOVA with Dunn's post hoc test	Figs. 7C and 8A

Supplementary Table 5. Predicted targets of MIR21-5p within the IL6/STAT3 gene family. A partial list of STAT3-related factors and receptors with their predicted or validated interaction with MIR21-5p according to TargetScan, RNA22, and miRdB prediction algorithms and the TarBase validated target database.

	TargetScan	RNA22	miRdB		TargetScan	RNA22	miRdB
IL6/STAT3 Family				Other STAT3-Associated Receptors			
Il6	-	+	-	*Cd4*	-	-	-
Il6ra	+	+	+	*Cd40*	-	-	-
Il6st	+	-	-	*Cd80*	-	-	-
Stat3	+	+	+	*Csf3r*	-	-	-
Lifr	+	-	-	*Cxcr4*	-	-	-
Cntfr	+	+	-	***Fas***	-	-	-
Lif	-	+	-	*Il18r*	-	-	-
Cntf	-	+	-	*Il1r1*	-	-	-
Osm	-	-	-	*Il2ra*	-	-	-
Osmr	-	-	-	***Tnfrsf10b***	-	+	-
Ctf	-	-	-	*Tnfrsf1a*	-	-	-
Clcf1	-	-	-	*Tnfrsf1b*	-	-	-
Il27	-	-	-				
Other STAT3 Activators							
Cxcl12	-	+	-				
Egfr	-	+	-				
Il10	-	-	-				
Il17a	-	-	-				
Il21	-	-	-				

Genes in bold are validated MIR21-5p targets from the TarBase v.8 database(Karagkouni *et al.*, n.d.) Predicted interactions using TargetScan v7.0(Agarwal *et al.*, 2015), RNA22(Miranda *et al.*, 2006), and miRdB(Wong and Wang, 2015) algorithms denoted +

Supplementary References

Agarwal V, Bell GW, Nam J-W, Bartel DP. Predicting effective microRNA target sites in mammalian mRNAs. Elife 2015; 4

Karagkouni D, Paraskevopoulou MD, Chatzopoulos S, Vlachos IS, Tastsoglou S, Kanellos I, et al. DIANA-TarBase v8: a decade-long collection of experimentally supported miRNA–gene interactions [Internet]. Available from: https://doi.org/10.1093/nar/gkx11415

Miranda KC, Huynh T, Tay Y, Ang Y-S, Tam W-L, Thomson AM, et al. A pattern-based method for the identification of MicroRNA binding sites and their corresponding heteroduplexes. Cell 2006; 126: 1203–17.

Obernosterer G, Martinez J, Alenius M. Locked nucleic acid-based in situ detection of microRNAs in mouse tissue sections. Nat Protoc 2007; 2: 1508–14.

Wong N, Wang X. miRDB: an online resource for microRNA target prediction and functional annotations. Nucleic Acids Res 2015; 43: D146-152.
