# Supplementary material for: Mir21 modulates inflammation and sensorimotor deficits in cervical myelopathy: data from humans and animal models
Source: Brain Commun. 2021 Jan 21;3(1):fcaa234. doi: 10.1093/braincomms/fcaa234 (PMC7878254; doi:10.1093/braincomms/fcaa234)
Supplement: fcaa234_Supplementary_Data [file fcaa234_supplementary_data.zip › Original Submission.pdf]

OXFORD UNIVERSITY PRESS | Brain Communications

MicroRNA-21 modulates inflammation and sensorimotor deficits in cervical myelopathy: evidence from humans and animal models

Journal:	*Brain Communications*
Manuscript ID	BRAINCOM-2020-248
Manuscript Type:	Original Article
Date Submitted by the Author:	23-Aug-2020
Complete List of Authors:	Laliberte, Alex; University of Toronto<br>Karadimas, Spyridon; University of Toronto<br>Vidal, Pia; University Health Network, Genetics and Development<br>Satkunendrarajah, Kajana; University Health Network, Genetics and Development<br>Fehlings, Michael; University of Toronto, Surgery
Keywords:	degenerative cervical myelopathy, spinal cord injury, biomarkers, neurodegeneration, microRNA-21, neuroinflammation

SCHOLARONE™
Manuscripts

MicroRNA-21 modulates inflammation and sensorimotor deficits in cervical myelopathy: evidence from humans and animal models

Authors: Alex M. Laliberte[1,2], Spyridon K. Karadimas[1,2,3], Pia M. Vidal[1], Kajana Satkunendrarajah[1], Michael G. Fehlings[1,2,3*]

Affiliations:

[1]Krembil Research Institute, University Health Network, Toronto, ON, Canada, M5T2S8.

[2]Institute of Medical Sciences, University of Toronto, Toronto, ON, Canada, M5T2S8.

[3]Division of Neurosurgery Spine Program, Department of Surgery, University of Toronto, ON, Canada, M5T2S8.

*To whom correspondence should be addressed: Michael G. Fehlings, Michael.Fehlings@uhn.ca, Krembil Discovery Tower, Toronto, ON, Canada, M5T2S8.

For Review Only

Abstract

Degenerative cervical myelopathy (DCM) is a common condition resulting from chronic compression of the spinal cord by degenerating structures of the spine. DCM patients present a wide range of outcomes, and the biological factors underlying this variability are poorly understood. Previous studies have found elevated microRNA-21 (miR-21) in the subacute and chronic neuroinflammatory environment following spinal cord injury. As chronic spinal cord neuroinflammation is a major feature of DCM, we hypothesized that miR-21 may be particularly relevant to DCM pathobiology, and could serve as a biomarker of disease progression. A prospective cohort study of 69 human DCM patients (36 male: 33 female) between the ages of 30 and 78 years was performed to identify the relationship between miR-21 expression, symptom severity, and treatment outcomes. Results from this study identified a positive correlation between elevated plasma miR-21 expression, initial symptom severity, and poor treatment outcomes. Subsequent validation of these relationships using a mouse model of DCM identified a similar elevation of miR-21 expression at 6 and 12 weeks after onset, corresponding to moderate to severe neurological deficits. To further determine how miR-21 affects DCM pathobiology, DCM was induced in a miR-21 knockout mouse line. Deletion of miR-21 preserved locomotor function on rotarod and forced swim tests, but also resulted in increased nociception based on tail flick, von Frey filament, and electrophysiological testing. Critically, miR-21 knockout DCM mice also had reduced spinal cord inflammation, demonstrated by the reduction of Iba1+ microglia by approximately 50% relative to wild type DCM controls. *In vitro* experiments using primary microglial cultures confirmed this relationship between miR-21 induction and microglial activation, as miR-21 expression was greatly increased following exposure to lipopolysaccharide (pro-inflammatory), Il4 (anti-inflammatory), and hypoxia. MiR-21 knockout did not appear to alter

For Review Only

the ability of microglia to respond to these stimuli, as expression of key pro- and anti-inflammatory response genes was not significantly altered. However, target prediction algorithms identified the IL-6/STAT3 pathway as a potential downstream target of miR-21, and subsequent *in vitro* testing found that expression of components of the IL-6 receptor complex, *Il6ra*, and *Il6st,* were significantly higher in miR-21 knockout microglia. In aggregate, these data show that miR-21 plays a role in the progression of motor deficits and neuroinflammatory modulation in DCM. Given this role in DCM neuroinflammation, and its association with poor patient outcomes, miR-21 represents a potential therapeutic target and a new marker for DCM patient prognostication.

For Review Only

Introduction

Degenerative cervical myelopathy (DCM) is a non-traumatic, chronic compressive form of spinal cord injury (SCI) that is considered to be the most common cause of spinal cord dysfunction worldwide (Nouri *et al.*, 2015). The symptoms of DCM are devastating and highly variable, including broad-based and unstable gait, loss of manual dexterity, pain, sensory loss, and spasticity (Nurick, 1972, Karadimas *et al.*, 2013*a*). Surgical decompression of the spinal cord can halt disease progression and can result in substantial neurological recovery, but a subset of DCM patients experience poor recovery, or even deteriorate further postoperatively (Fehlings *et al.*, 2015, Karadimas *et al.*, 2015*b*). Unfortunately, the underlying mechanisms that result in this variability in patient outcomes are either unknown or not detectable with existing tools, making accurate prognosis and optimal management of DCM a significant challenge. New insights into the underlying molecular mechanisms of DCM, and the development of relevant biomarkers are required to address this critical problem.

Circulating microRNAs have recently emerged as powerful biomarkers for SCI and numerous other diseases. Also, as regulators of mammalian transcription, disease-related microRNAs have potential as therapeutic targets. As such, our goal was to identify a microRNA biomarker with mechanistic links to DCM. DCM differs from SCI due to the chronic and progressive nature of spinal cord compression, with chronic ischemia (Breig *et al.*, 1966, Karadimas *et al.*, 2015*b*; Vidal *et al.*, 2017) and chronic inflammation (Yu *et al.*, 2011, Karadimas *et al.*, 2015*a*) being dominant mechanisms. Following SCI, microRNA-21 (miR-21) is one of few microRNAs to have elevated expression beyond the subacute phase (Bhalala *et al.*, 2012), making it a possible mediator of chronic spinal cord inflammation. MiR-21 is one of the most commonly upregulated genes in central nervous system (CNS) injuries (Liu *et al.*, 2009; Bhalala *et al.*, 2012; Yunta *et al.*, 2012; Karl *et al.*, 2017; Leinders *et al.*, 2017), and its overexpression has been

For Review Only

observed in neuroinflammatory (Bergman *et al.*, 2013; Murugaiyan *et al.*, 2015; Harrison *et al.*, 2016) and hypoxic/ischemic states (Ziu *et al.*, 2011; Cui *et al.*, 2017; Jia *et al.*, 2017).

Given the suspected role of miR-21 in hypoxia, inflammation, and its overexpression in the chronic phase of SCI, we hypothesized that miR-21 would be induced by the chronic spinal cord compression of DCM. Further, given that miR-21 has been implicated in inflammatory gene regulation (Bergman *et al.*, 2013; Wang *et al.*, 2015), we postulated that manipulation of miR-21 could significantly reduce the extent of harmful neuroinflammation and neurological deficits observed in DCM animals. Herein, we demonstrate that miR-21 is associated with more severe presentation and worse treatment outcome in a prospective clinical study of DCM patients. This relationship was further supported by miR-21 upregulation in a DCM mouse model, and the observation that deletion of miR-21 results in significant preservation of motor function. Finally, we also demonstrate that miR-21 is upregulated during microglial activation, and that loss of miR-21 significantly reduces the number of Iba1+ microglia in the compressed spinal cord. In aggregate, these animal and human patient data implicate miR-21 as a critical regulator of neuroinflammation and cervical myelopathy pathology, suggesting potential utility of miR-21 as a prognostic biomarker and therapeutic target for cervical myelopathy.

For Review Only

Materials and Methods

Human Plasma microRNA Study

Study design, data collection and blood analysis protocols were all approved by the University Health Network Research Ethics Board, and consent was obtained for all participants in this study prior to enrolment. Subjects were enrolled at the neurosurgical clinic at the Toronto Western Hospital. Inclusion criteria required subjects to be English speakers between 18-80 years

of age, with imaging confirmation of cervical cord compression and at least one of the following neurological signs: corticospinal motor deficits, atrophy of intrinsic hand muscles, hyperreflexia, positive Hoffman's sign, upgoing plantar responses, lower-limb spasticity, broad-based unstable gait. Exclusion criteria included: previous spine surgery, symptomatic lumbar stenosis, symptoms due to trauma, uncontrolled or insulin-dependent diabetes, neutropenia, creatinine>1.2mg/dL, liver enzymes (ALT or AST) 3 times higher than normal, systemic infection (HIV, hepatitis, etc), history of hypertension, active malignancy (within the past 5 years), recent history of substance abuse (within past 3 years), and history of myocardial infarction, stroke, or heart failure. Initially, 88 DCM subjects at the Toronto Western Hospital volunteered, with 69 meeting study criteria. These patients were between the ages of 30 and 78 years, with a mean age of 56.0 ± 10.6 (SD) years at the time of enrolment. Quantitation of miR-21 from the 69 DCM patients (36 Male: 33 Female) was performed blinded to subject identity or any other clinical data. Severity of DCM was quantified using the modified Japanese Orthopedic Association (mJOA) scale. The mJOA is a multifaceted assessment tool that grades the impairment caused by upper motor, lower motor, sensory and autonomic dysfunction in DCM patients, providing an overall score out of 18 points (Benzel *et al.*, 1991; Tetreault *et al.*, 2017). Blood was collected following the neurological assessment and prior to any surgical intervention or treatment. Follow-up assessment of DCM subjects occurred one year after surgery or the initial assessment. Of the 43 subjects given surgery, 8 were lost to follow-up, (unreachable, or cited lack of transportation during the study period). Subjects lost to follow up were excluded from analyses examining patient outcome.

Animals

For Review Only

All protocols involving animal use were designed and carried out in accordance with Canadian Council for Animal Care guidelines with ethical approval granted by the institutional animal ethics review board of the University Health Network. All animals were provided standard chow and water *ad libitum* and housed in a barrier containment facility with a 12:12 light/dark cycle. Mice used in this study were female between 8-12 weeks old at the beginning of experiments. Mouse strains used in this study were commercially available: wild type (C57bl/6 - Jackson Labs# 000664) and miR-21 knockout (Jackson Labs# 016856). A total of 55 mice were used, not including those used to derive primary microglial cultures.

Experimental Design

Sample sizes were estimated based on α=0.05, and a minimum power of 0.80 to detect effect sizes observed in comparable experiments from our laboratory. Mice of each genotype were allocated to either DCM or sham groups on an alternating basis. DCM and sham surgeries were performed as previously described (Karadimas *et al.*, 2013*b*; Vidal *et al.*, 2017). A complete description of the surgery can be found in the supplementary material. Following surgical induction of DCM, mice were assigned new identifiers by a third party to blind experimenters to animal genotype. Order of behavioural testing was randomized throughout, and unblinding occurred at the experimental endpoint.

Sample Collection and Tissue Processing

At the experimental endpoint, mice were euthanized via isoflurane overdose. For RNA quantification experiments, blood was collected from the left ventricle into EDTA coated

For Review Only

vacutainers (BD Biosciences, Mississauga, Canada) and centrifuged at 1500 x g for 10 minutes to separate plasma from the cellular pellet. Plasma was then aliquoted and snap frozen in liquid nitrogen prior to storage at -80°C. Mice were transcardially perfused with 1X PBS to clear tissues of blood, and the spinal cords were extracted, frozen on liquid nitrogen and stored at -80°C. For immunohistochemistry, mice were transcardially perfused with 1X PBS, then with ice cold 4% (w/v) Paraformaldehyde (PFA) in 1X PBS. Spinal cords were post-fixed in 4% PFA with 10 % (w/v) sucrose for 8-16 hours at 4°C, followed by a PBS wash and overnight cryoprotection in a 30% (w/v) sucrose 1X PBS solution. Spinal cords were then embedded in Shandon™ M-1 embedding matrix (ThermoFisher Scientific, Waltham, USA), sliced into 30 µm cross-sections on a cryostat (Leica Microsystems Canada, Richmond Hill), and mounted on Superfrost Plus microscope slides (ThermoFisher Scientific, Waltham, USA).

RNA Extraction

RNA extraction from frozen tissue samples and fresh microglial pellets was performed using the miRCURY cell and plant RNA isolation kit (Exiqon, Vedbaek, Denmark), with supplemental lysis additive for the spinal cord tissue, according to the manufacturer's instructions. Briefly, 3 mm spinal cord segments were crushed under liquid-nitrogen using a dry ice-cooled mortar and pestle. The recovered powder was then dissolved in lysis solution and passed through a 25 gauge needle to break up remaining tissue and aid in cell lysis prior to beginning the RNA isolation protocol. Eluted RNA quantity and purity was determined using a NanoDrop 1000 spectrophotometer (ThermoFisher Scientific, Waltham, USA) and a BioAnalyzer system (Agilent Technologies, Santa Clara, USA). For the isolation of total RNA from plasma samples, extraction

For Review Only

was performed using the miRCURY biofluids RNA kit (Exiqon, Vedbaek, Denmark) according to the manufacturer's instructions.

cDNA synthesis

cDNA synthesis for microRNA quantification was performed using the Universal cDNA Synthesis kit II (Exiqon, Vedbaek, Denmark) according to the manufacturer's instructions. cDNA synthesis for mRNA transcripts was performed using the High-Capacity RNA-to-cDNA™ Kit (ThermoFisher Scientific, Waltham, USA) according to the manufacturer's instructions. Following cDNA synthesis, reactions were cooled to 4˚C for immediate use, or stored at -20˚C.

Real-Time Quantitative Polymerase Chain Reaction

Quantitation of miR-21 was performed using ExiLENT SYBr®-green-based rt-qPCR with locked nucleic acid (LNA) probes specific to the mature sequence of miR-21 (Exiqon, Vedbaek, Denmark). Human plasma miR-21 expression was normalized to miR-423-5p. Relative differences of miR-21 between groups was determined using the ΔΔCt method after normalization to endogenous controls, spliceosomal RNA U6 (spinal cord tissue) or miR-16 (mouse plasma). For the quantitation of mRNA transcripts (*Tnfa*, *Nos2*, *Arg1*, *Il6*, *Il6ra*, *Il6st*), rt-qPCR was performed using specific Taqman™ probes and Taqman™ Fast Advanced master mix (ThermoFisher Scientific, Waltham, USA). Relative quantities were determined using the ΔΔCt method after normalization to endogenous control, *Gapdh*. All reactions were performed in triplicate and analyzed using a 7900HT rtPCR system (ThermoFisher Scientific, Waltham, USA) using the recommended cycling parameters for the qPCR master mix.

For Review Only

Rotarod Test

Mice were placed on a rotating cylinder suspended at a height that is high enough to induce fall avoidance, but low enough to prevent injury. Prior to data collection, mice were trained at low speed for a total of 30 minutes/day for 5 days to gain task-specific competency and increase intra-animal reproducibility. During testing, rotation of the cylinder was gradually increased from 3.5 to 35 rpm. Both the total time spent on the rotarod, and the final speed of the mouse were recorded following a fall from the device. Three rotarod runs were collected for each animal and averaged. Sham-operated animals, lacking noticeable locomotor deficits, did not fall from the rotarod following training, and were therefore assigned max values of 35 rpm and 500 s. Since the competency training required for the rotarod test could be considered a form of physical therapy, it was only performed at the experimental endpoint, 12 weeks, to avoid potential influence on the progression of neurological deficits in the DCM mouse model.

For Review Only

Catwalk Gait Analysis

Analysis of spatiotemporal gait parameters was performed using the Catwalk XT walkway system (Noldus, Leesburg, USA) as previously described (Karadimas *et al.*, 2013*b*; Vidal *et al.*, 2017). Briefly, mice were recorded while moving across the Catwalk XT walkway. Only uninterrupted runs with a minimum of 3 continuous step cycles were considered for analysis. Compliant runs were analyzed and averaged for each animal.

Von Frey Test

Sensitivity to mechanical stimulus was assessed in mice using Von Frey monofilaments via the frequency method (Bourquin *et al.*, 2006). Mice were allocated to individual 15 x 15 cm chambers set on a 0.3 x 0.3 cm grate (allowing access to the inferior surface of paws from below) and allowed 1 hour to acclimatize. Each forepaw and hindpaw was assessed ten times at each experimental timepoint by contacting the pad of paw with the 0.4g Von Frey hair, producing either a negative (no withdrawal) or positive (withdrawal) response. The total number of positive responses for each paw were tallied and represented as an average value out of 10. Pre-surgical assessment of animals was performed to identify and exclude animals with abnormally high withdrawal responses (>2) resulting in 1 mouse of each genotype being excluded from pain testing. Mice were given ample time between trials to avoid animal stress and irritation of the target site. Data collection was performed at the same time of day, by the same experimenter.

Tail Flick Test

Sensitivity to thermal stimulus was assessed in mice using the tail flick test. Mice were held on the Tail Flick Analgesia Meter (IITC Life Sciences, Woodland Hills, USA) with the tail exposed. The heating lamp was initialized for each trial and the time to tail withdrawal was automatically detected. Three trials were performed for each animal at each timepoint, and the mean value was used for statistical analysis.

Lumbar Dorsal Horn Local Field Recordings

Mice were anesthetized using 1% isoflurane and placed prone on a stereotaxic frame to expose the dorsal aspect of the hindlimb. Physiological body temperature was maintained using a

For Review Only

heating pad. The sural nerve was dissected in the left hind limb from its origin from the sciatic nerve to the lateral malleolus and the intact sural nerve in the popliteal fossa was electrically stimulated with bipolar silver hook electrodes placed underneath the nerve. The nerve from each animal was covered in mineral oil and stimulated with square-wave pulses of 0.04 msec duration at a frequency of 0.13 msec. The stimulation was initiated first at a minimal current strength required to elicit extracellular potential from the dorsal surface of the lumbar spinal cord (L4) (0.1-0.5 μA). Extracellular potentials were recorded from the dorsal surface of the lumbar spinal cord (L4) with tungsten microelectrodes using Keypoint Portable (Dantec Biomed, Denmark). At a bandwidth of 10 to 3000Hz, a total of 100 SEPs were averaged. The experiment was repeated three more times with incremental increases in current strengths by 0.1 μA.

Forced Swim Test

Mice were assessed for motor deficits in a sensory-reduced environment using a modified version of the Schnell swim test (Broggini *et al.*, 2016). Individual mice conducted multiple swim trials in a Plexiglas basin containing 25-30˚C water and were recorded using a PROMON 501 high-speed camera (AOSTechnologies, Baden, Switzerland) at 50 frames per second. Average swim speed was calculated by measuring the time elapsed to swim a known distance. Only swim trials with continuous swimming (without animals contacting the sides of the basin) were analyzed.

For Review Only

Immunohistochemistry and Stereological Counting

Microglia and recruited spinal cord macrophages were detected via immunohistochemistry using an anti-Iba1 rabbit polyclonal antibody (1:500, Wako Life Sciences, Mountain View, USA),

as previously described (Yu *et al.*, 2011). Fluorescence visualization of Iba1 was enabled via AlexaFluor594-conjugated anti-rabbit IgG (1:400, Sigma-Aldrich, St Louis, USA) and nuclear fluorescence was provided by 4',6-diamidino-2-phenylindole (DAPI, Vector Laboratories, Burlingame, USA). Iba1-positive, DAPI positive cells were estimated throughout the compressed region of the cervical spinal cord using the optical fractionator probe in Stereoinvestigator (MBF Bioscience, Williston, USA).

Microglial Primary Cell Culture

Mixed glial cultures were prepared from P0-P2 aged pups from C57 or miR-21 KO breeding pairs as described by others (Vidal *et al.*, 2013; Lam *et al.*, 2017). A more detailed description of the protocol can be found in the supplementary material. All FBS used for culture experiments was depleted of exosomes by overnight ultracentrifugation at 120,000 x g using a 70Ti rotor in an Optima L-80 preparative ultracentrifuge (Beckman-Coulter, Brea, USA). To induce pro-inflammatory and anti-inflammatory activation states, 10 ng/mL E.coli Lipopolysaccharide (LPS) (Sigma-Aldrich, St Louis, USA) or 20 ng/mL IL-4 (R&D Systems Inc., Minneapolis, USA) was added to the microglial culture, respectively. Hypoxia was induced by culturing microglia for 24 hours at 1% O_2 in a Hypoxia CO_2 incubator (Nuaire, Plymouth, USA). All microglial treatments were harvested after 24 hours, gently washed with 1X PBS, and processed for RNA extraction.

Statistical Analysis

For Review Only

The correlation between miR-21 and the mJOA score was calculated using the Pearson correlation test (Fig. 1B-C). Expression of miR-21 in DCM mouse spinal cords and plasma, as well as in primary microglia were analyzed by one-way ANOVA with Dunnett's post-hoc test (Figs. 2B and 7A). Rotarod data and Iba1 cell counts were analyzed with independent samples t-tests (Figs. 3A and 6B). Catwalk, von Frey, and tail flick tests were analyzed using repeated measures ANOVA with Sidak's post-hoc test (Figs 3C, 4A, and 4B). Swim speed was analyzed using one-way ANOVA with Tukey's post-hoc test. Gene expression in WT and miR-21 KO microglial cells was analyzed using two-way ANOVA with Dunn's post-hoc test (Figs. 7C and 8A). ANOVA assumptions of equality of variance were tested using the Brown-Forsythe test. Data are presented as group means ± SEM. All graphs and statistics were created using Prism 6 (GraphPad Software Inc, La Jolla, USA) and SPSS v22 (IBM, Armonk, USA).

Data availability

The data that support the findings of this study are available from the corresponding author, upon reasonable request

Results

miR-21 expression in human DCM patient plasma correlates with increased symptom severity and worse treatment outcomes

To examine whether levels of miR-21 were relevant to DCM pathobiology in humans, circulating levels of miR-21 were examined in the plasma of 69 DCM patients and compared to the extent of neurological deficits (Fig 1A). In total, 41 DCM subjects (21 Male: 20 Female) with

For Review Only

mild symptoms (mJOA≥15), and 28 subjects (15 Male: 13 Female) with moderate to severe neurological deficits (mJOA<15) were examined. Normalized levels of miR-21, denoted as dCt values (where -1 dCt represents a doubling of expression), demonstrated a modest correlation to the modified Japanese Orthopedic Association scale (Fig. 1Bi, Pearson correlation test, r=0.238, p=0.049, n=69), the directionality of which indicating that greater expression of miR-21 correlates with a greater extent of neurological deficits. This correlation was substantially stronger among individuals with moderate to severe deficits (Fig 1Bii, r=0.424, p=0.025, n=28), than in the mild cohort, where no significant correlation was observed (r=-0.016, p=0.923, n=41).

To evaluate the potential of miR-21 in predicting patient outcomes, DCM subjects scheduled for decompressive surgery were assessed 1 year after treatment using the mJOA scale. Within this patient cohort, greater pre-operative miR-21 expression was correlated with worse patient mJOA scores after surgery (Fig 1C, r=0.368, p=0.030, n=35), suggesting that elevated levels of miR-21 may be detrimental, or indicative of poor outcome for human DCM patients undergoing surgical treatment.

miR-21 is upregulated systemically and in the compressed spinal cords of DCM mice

As a means of further examining the observed relationship between miR-21 expression and DCM severity, expression of miR-21 was determined in a mouse model of DCM (Karadimas *et al.*, 2013*b*; Vidal *et al.*, 2017). This DCM mouse model entails the insertion of a progressively ossifying biomaterial into the spinal canal, causing gradual spinal cord compression. Expression of miR-21 was determined 3, 6, and 12 weeks after material implantation and compared to age-matched naïve controls (Fig. 2A) for both plasma and spinal cord samples from the compressed cervical region. As expected, levels of miR-21 were elevated in the spinal cords of DCM mice

For Review Only

compared to uninjured mice. Mirroring the human data, miR-21 expression was greatest in animals with moderate to severe deficits, showing significant upregulation in the 6 and 12 week cohorts (Fig. 2B, ANOVA, $p<0.0001$, Dunnett's post hoc, $q=8.002$ and 3.54, respectively, $df=12$). This increase in miR-21 expression at 6 and 12 weeks was also observed systemically in the plasma of DCM mice (Fig. 2B, ANOVA, $p<0.0001$, Dunnett's post-hoc, $q=4.272$ and 7.453, respectively, $df=12$). As a secondary means of confirming miR-21 expression in the compressed spinal cord, in situ hybridization for miR-21 was also performed on spinal cord tissue sections using a miR-21-specific Locked Nucleic Acid (LNA) probe. Development of mir-21 probed samples revealed strong precipitate formation in the spinal cord parenchyma of DCM, but not a miR-21 knockout DCM mouse, demonstrating specificity of the miR-21 staining within the DCM spinal cord (Fig. S1).

Deletion of miR-21 improves locomotor function but with evidence of an antalgic gait pattern

To determine whether miR-21 influences the underlying pathology and the neurological deficits associated with DCM, we examined whether the development of locomotor deficits, one of the cardinal symptoms of human DCM, was significantly altered in miR-21 knockout mice. First, we examined the locomotor performance of WT and miR-21 KO mice using the rotarod test. All sham-operated animals from both WT and miR-21 KO groups were able to remain on the rotarod at maximum speed and for the entire duration of the test (500 seconds) after the training period. Among the DCM mice, wild-type animals, on average, were able to maintain balance on the rotarod for 220.2 ± 21.36 (SEM) seconds, and had a maximum speed of 25.96 ± 1.587 (SEM) rpm (Fig. 3A). In contrast, miR-21 knockout DCM animals ran approximately 70% longer than WT ($p=0.004$, $t=3.547$, $df=12$), at 376.1 ± 38.40 (SEM) seconds, and reached an average top speed

For Review Only

of 33.21 ± 0.8473 (SEM) rpm, approximately 28% faster than WT DCM animals ($p=0.0017$, $t=4.031$, $df=12$, Fig 3A).

While rotarod experiments demonstrated significant preservation of locomotor function resulting from miR-21 deletion, analyses of spontaneous locomotor gait in these animals revealed evidence of gait disruption (Fig. 3B). Specifically, repeated measures ANOVA of Catwalk gait results identified significant decreases in forelimb stride length at 2 weeks (Sidak's test, $p=0.0135$, $t=3.083$, $DF=48$) and 6 weeks (Sidak's test, $p=0.0333$, $t=2.747$, $DF=48$), and decreases in hindlimb stride length at 2 weeks (Sidak's test, $p=0.0257$ $t=2.846$, $DF=48$), 6 weeks (Sidak's test, $p=0.0114$, $t=3.144$, $DF=48$), and 12 weeks (Sidak's test, $p=0.0437$, $t=2.641$, $DF=48$) in miR-21 KO DCM mice compared to WT DCM mice (Fig. 3C). Animal miR-21 genotype was identified as a significant factor for hindlimb swing speed (repeated measures ANOVA, $F=7.658$, $DF=1,12$, $p=0.017$), but multiple comparisons testing did not identify specific differences at any of the time points tested. Finally, forepaw and hindpaw base of support were both significantly increased in miR-21 KO DCM mice at 2 weeks (Sidak's test, $p=0.0021$, $t=3.722$, $DF=48$, and $p=0.0036$, $t=3.539$, $DF=48$, respectively). The Catwalk and rotarod data appear contradictory; however, several of the gait parameters identified by the Catwalk system are similarly affected in models of axial or neuropathic pain, common symptoms of DCM (Miyagi *et al.*, 2013; Kameda *et al.*, 2017).

Sensory assessments demonstrate increased sensitivity to noxious stimuli in miR-21 knockout mice

Therefore, to identify whether loss of miR-21 altered the manifestation of neuropathic pain in DCM, animals were assessed for mechanical allodynia using the Von Frey test and thermal hyperalgesia using the tail flick test. Using the frequency method, Von Frey filament testing revealed a significant increase in withdrawal frequency in forepaws at 8 weeks, 10 weeks and 12

For Review Only

weeks (Fig. 4A, Repeated Measures ANOVA, Sidak's post-hoc, p=0.0005,t=4.212; p=0.0168, t=3.12; and p=0.0065, t=3.432, respectively, df=60) and in hindpaws at weeks 10 and 12 (Repeated Measures ANOVA, Sidak's post-hoc, p=0.0004, t=4.323; and p=0.0015, t=3.891, respectively, DF=60). In general, miR-21 KO DCM animals demonstrated both an earlier onset, and a greater overall increase in the frequency of paw withdrawal, indicating a greater magnitude of hypersensitivity than in age-matched WT DCM mice. Results from the tail flick test supported this finding, as the miR-21 KO DCM mice showed a more rapid withdrawal latency than WT DCM, specifically in the early progression of the disease at 2 week timepoint (Fig. 4B, Repeated Measures ANOVA, Sidak's post-hoc, p=0.0382, t=2.862, DF=70)).

While these behavioural responses show that sensory stimulation induces a greater effect in miR-21 KO DCM animals, both the von Frey and tail flick tests rely on a motor response to signal a pain response. As such, differences in motor deficits may confound the nature of the sensory differences between the two genotypes. To distinguish between motor and sensory components of these behavioural tests, we performed *in vivo* electrophysiology in miR-21 KO mice, and WT DCM and sham mice. Stimulation of the sensory aspect of the sural nerve was performed while recording local field potentials from the dorsal horns of the spinal cord at L4. The minimum stimulation threshold to induce dorsal horn field potentials for both miR-21 KO and WT DCM mice were reduced relative to sham animals (ANOVA, Tukey's post-hoc, p=0.0067, q=6.056 and p=0.029, q=4.569, respectively, DF=8), suggesting sensory allodynia, but were not significantly different between WT and miR-21 KO mice. However, the amplitude of the evoked response in the miR-21 KO DCM animals was significantly increased relative to WT DCM mice, indicating a greater extent of sensory sensitivity in DCM mice lacking miR-21 (Repeated Measures ANOVA, Tukey's post-hoc, p=0.008, and p=0.001, respectively, DF=8).

For Review Only

miR-21 KO DCM mice outperform wild type DCM cohort in swimming task that limits mechanosensory feedback

Given that abnormal nociception in miR-21 KO DCM mice could be confounding their motor output in volitional motor tasks, a swim test was devised as a method to assess locomotor function while minimizing mechanosensory feedback that could be perceived as pain in the DCM mice. This, in theory, would partially isolate the effects of miR-21 deletion on motor function from those associated with abnormal pain sensation. During the swim test, miR-21 KO DCM mice swam at an average speed of 14.79 cm/s, a speed that was similar to age-matched sham values (Fig. 5A-B). In contrast, WT DCM mice were significantly slower (9.29 cm/s, Tukey's test, $p=0.0048$, $q=5.873$, $df=14$) than miR-21 KO DCM animals during the swimming task (Fig. 5B), providing support for the observed improvement in motor preservation in miR-21 knockout mice.

Spinal cord sections show reduced microglial recruitment in miR-21 KO DCM mice

Based on the differences in motor and sensory phenotype between WT and miR-21 KO DCM mice, we set out to identify differences in spinal cord pathology that might account for these disparities. Since miR-21 has been implicated in neuroinflammation (Bergman *et al.*, 2013; Wang *et al.*, 2015), and studies from our laboratory and others have implicated microglia/macrophages as critical mediators of inflammation in DCM models (Yu *et al.*, 2011; Hirai *et al.*, 2013; Moon *et al.*, 2014), we hypothesized that miR-21 may affect the microglia/macrophage inflammatory response. Unbiased stereological counting using the Stereoinvestigator Optical Fractionator probe was employed to compare the number of Iba1+ microglial cells in the compressed regions of miR-

21 KO DCM and WT DCM spinal cords (Fig. 6A). This experiment found that the number of Iba1+ microglia throughout the compressed region of the spinal cord was substantially decreased in miR-21 KO mice, with microglial counts totaling less than 50% of the WT DCM values (Fig. 6B, t-test, $p=0.0006$, $t=4.785$, $df=11$).

miR-21 is induced in microglial cells during activation and hypoxia

The reduction in recruited microglia in miR-21-deficient mice suggested that miR-21 potentially plays a role in the inflammatory activation of microglia in DCM. To determine whether miR-21 is related to the activation of microglia, we isolated primary microglia from the cortex of P0-P2 mice and treated them with either LPS to induce a classical pro-inflammatory phenotype, IL-4 to induce an M2 alternative activation state, or hypoxia (1% O_2) for 24 hours. All cell conditions demonstrated a significant increase in miR-21 relative to control, with the greatest increases resulting from LPS (ANOVA, Dunnett's post-hoc, $p=0.0006$, $q=6.293$, $DF=8$) and hypoxia treatments (ANOVA, Dunnett's post-hoc, $p<0.0001$, $q=14.73$, $DF=8$), with relative increases of approximately 8 and 20-fold, respectively (Fig. 7A).

miR-21 knockout does not impair microglial activation or polarization

To determine whether miR-21 is necessary for microglial polarization or viability, primary microglia were also isolated from P0-P2 miR-21 KO pups. Microglial yield from miR-21 KO was similar to wild type, with three P2 pups yielding between 4-5x10^5 microglial cells in all primary microglial isolations, thus suggesting that viability was not substantially affected by miR-21 deletion. Characterization of the microglia did not reveal any obvious abnormalities in cell morphology, and additions of pro-inflammatory treatments produced the expected morphological

For Review Only

changes (Fig. 7B), specifically the retraction of processes and the adoption of an amoeboid migratory mode. With respect to the inflammatory phenotype of the microglia, expression of M1 macrophage polarization markers including Nos2 and Tnfα were not significantly different between wild-type and miR-21 KO microglia in either the resting state or following induction with LPS (Fig. 7C). Alternative activation (M2) marker Arg1, was moderately lower in the untreated miR-21 microglia (two-way ANOVA, Sidak's post-hoc, $p=0.002$, $t=4.787$, $DF=9$), but induction of the M2 phenotype with IL-4 did not result in significant differences in Arg1 between the two genotypes (Fig. 7C). As such, it appeared as though the loss of miR-21 did not significantly impact the ability of the microglia to respond to pro- and anti-inflammatory cues.

miR-21 knockout alters IL-6 signal transducer expression during microglial inflammatory challenge

To determine how the loss of miR-21 might affect microglial function, we performed a predictive target analysis using a number of target prediction algorithms (TargetScan, RNA22, and miRdB) and a validated target database (TarBase). Aggregation of the results of these analyses revealed a number of predicted targets for miR-21 in the IL-6/STAT3 pathway, a significant signaling pathway involved in both inflammation (Yasukawa *et al.*, 2003; Fernando *et al.*, 2014) and neuropathic pain (Dominguez *et al.*, 2008; Hori *et al.*, 2016; Molet *et al.*, 2016) (Table S1). We therefore examined the mRNA expression of three of the predicted targets (Il6, Il6ra, and Il6st) in both the quiescent and LPS-activated states of wild-type and miR-21 KO microglia. For the predicted targets, Il6, Il6ra, and Il6st, miR-21 genotype was a significant factor in the analysis (two-way ANOVA, $p=0.0112$, $p=0.0190$, $p=0.0034$, respectively). Multiple comparison testing identified Il6 and Il6st as significantly higher in LPS-treated miR-21 KO microglia (Fig. 8, Sidak's

For Review Only

post-hoc, p=0.0469, t=2.623; p=0.0095, t=3.257, respectively, DF=11), while Il6ra was higher in the untreated miR-21 KO microglial cultures (Sidak's post-hoc, p=0.0023, t=4.357, DF=11).

Discussion

Due to the gradual and variable nature of DCM development, the molecular determinants of DCM pathobiology have largely remained elusive, yielding few molecular biomarkers or therapeutic targets. Using the combination of prospective human data, a DCM animal model, and miR-21 knockout mice, this study demonstrated a link between miR-21, DCM symptom severity and progression, and microglial inflammation in the compressed spinal cord. Given the known relevance of microglial inflammation to DCM pathobiology (Yu *et al.*, 2011; Hirai *et al.*, 2013; Takeura *et al.*, 2019), we posited that miR-21's relationship to DCM motor deficits could be mediated through microglia. Subsequent *in vitro* experiments confirmed the induction of miR-21 during microglial activation, and identified the Il6/STAT3 pathway as a potential target for miR-21's effect on microglia. While further research will likely be required to determine the relevance of the proposed miR-21/Il6/STAT3 molecular pathway to DCM pathobiology, this study nonetheless suggests the importance of miR-21 in DCM progression and suggests a plausible microglial-dependent mechanism for this effect.

The precise role of miR-21 in inflammation remains an area of controversy, as several studies have identified its expression under pro- or anti-inflammatory conditions (reviewed in Gaudet *et al.*, 2017). Interestingly, Il6 signaling has a similarly complex and context-dependent role in pro-inflammatory (Romano *et al.*, 1997; McLoughlin *et al.*, 2005; Fielding *et al.*, 2008) and anti-inflammatory mechanisms. Within macrophages, Il6/STAT3 signaling has been associated with the enhancement of M2 anti-inflammatory polarization (Yasukawa *et al.*, 2003; Fernando *et al.*, 2014). Manipulation of miR-21 expression in macrophages is consistent with antagonism of

For Review Only

Il6/STAT3, as miR-21 expression inhibits M2 anti-inflammatory polarization, and miR-21 knockout rescues M2 polarization and increases STAT3 activation (Wang *et al.*, 2015), suggesting a pro-inflammatory role of miR-21 in macrophages. Consistent with that conclusion, our results demonstrate a reduction of Iba1+ microglia/macrophages in miR-21 KO DCM mice.

Despite the noted decrease in Iba1+ microglia, miR-21 KO mice experienced a greater degree of mechanical and thermal hypersensitivity than wild-type DCM animals. A potential effect of miR-21 on nociception was anticipated, as some recent studies have identified increased levels of mir-21 in neuropathic pain states (Hori *et al.*, 2016; Karl *et al.*, 2017; Leinders *et al.*, 2017; Zhong *et al.*, 2019). However, microglia have well-established roles in the genesis of neuropathic pain, and a reduction in local microglia accompanied by increased pain sensitivity was unexpected. While further research will be required to determine the precise mechanism for this increase in pain sensitivity, the proposed role of miR-21 in the regulation of the Il6/STAT3 pathway could potentially influence this phenomenon. Intrathecal administration of IL-6 is sufficient to generate mechanical allodynia (DeLeo *et al.*, 1996), and it has previously been demonstrated that both microglial and astrocyte STAT3 activation is central to neuropathic pain (Dominguez *et al.*, 2008, 2010; Tsuda *et al.*, 2011; Liu *et al.*, 2013). Microglial STAT3 activation is after rat peripheral nerve injury (Dominguez *et al.*, 2008), and IL6 knockout mice do not develop neuropathic pain, nor the expected increase of miR-21 after injury (Hori *et al.*, 2016), suggesting a possible reciprocal relationship between miR-21 and Il6/STAT3 signaling. In line with these studies and the proposed inhibitory relationship of miR-21 on IL6/STAT3 signaling, our results support an antagonistic role of miR-21 in the development of neuropathic pain.

Given that miR-21 appears related to the underlying pro-inflammatory signaling in DCM, miR-21 could represent a useful biomarker for the clinical DCM population. The extent of

For Review Only

compression determined from spinal cord imaging is an insufficient predictor of future neurological deficits, particularly in mild or non-myelopathic cases (Oshima *et al.*, 2012; Li *et al.*, 2014; Kovalova *et al.*, 2016). While miR-21 expression in the human cohort was more strongly correlated to severe cases, it is possible that outliers with high miR-21 expression in the mild patient group may be those with greater risk of imminent deterioration. This information would be extremely valuable for clinical decision-making and the prioritization of surgical intervention, but additional longitudinal studies of early-stage/mild DCM patients will be required to evaluate miR-21's utility for this application. However, based on the existing data, it appears that high miR-21 expression is related to worse patient outcomes following surgical intervention. This prognostic information may provide the incentive to develop complementary treatment strategies for individuals with lower potential for recovery. Further validation of miR-21's relationship to poor surgical outcomes, and determination of its responsiveness to anti-inflammatory treatment would be valuable in determining its potential utility as a DCM clinical biomarker.

While this work underlines a previously unknown role of miR-21 in DCM pathobiology, there are some caveats that limit the interpretation of our results. To constrain the scope of this research, we primarily focused on the mechanism of miR-21 in microglia/macrophages. However, miR-21 has also been implicated in the function of other cells types in neurological injury models. In spinal cord injury, miR-21 is reported to play a role in the activation of reactive astrocytes, astrocyte hypertrophy and glial scar formation (Bhalala *et al.*, 2012). Other studies have also reported anti-apoptotic function of miR-21 in neurons after spinal cord (Hu *et al.*, 2013) and brain injury (Ge *et al.*, 2014), while another has implicated miR-21 binding to TLR-7 as a potential mediator of pro-inflammatory neuron apoptosis (Yelamanchili *et al.*, 2015). Given the diverse mechanisms described, we acknowledge that miR-21 potentially has multiple roles depending on

For Review Only

the pathological context, and therefore recommend a relatively narrow interpretation of these results in the context of non-traumatic, compressive CNS injury. Also, while our data indicate a regulatory role for miR-21 in the IL-6 signaling pathway, the current study does not directly examine the downstream consequences of Il6, Il6ra, and Il6st de-repression, nor does it exclude the involvement of other signaling pathways relevant to microglial function. Future investigations will be required to further examine the proposed link between miR-21/Il6/STAT3 interactions and microglial function in DCM.

In conclusion, the results from both human and animal experiments suggest that miR-21 is implicated in the development of neurological deficits in DCM, likely via a pro-inflammatory mechanism. This observed relationship between miR-21 expression and patient outcomes should provide useful prognostic information and mechanistic insights for future studies of DCM and justifies further examination of miR-21 as a potential biomarker of disease progression.

Acknowledgments: Thanks to TJ Siddiqui and Lyanne Schlichter for assistance with primary microglia culture protocols. **Funding**: Funding was provided through a CIHR Canada Graduate Scholarship (AML), the Ontario Graduate Scholarship program (AML) and grants from the Canadian Institutes of Health Research (MGF), the Cervical Spine Research Society, and the Physicians' Services Incorporated Foundation (MGF). **Competing interests**: The authors report no competing interests. **Author contributions**: Conceptualization of Study – AML, SKK, MGF. Experimental Design – AML, SKK, KS, MGF. Data Collection – AML, SKK, PV, KS. Writing and Editing of Manuscript – AML, SKK, PV, KS, MGF.

For Review Only

Supplementary Materials:

Supplementary Materials and Methods

DCM and Sham Surgical Protocols

Briefly, animals were pretreated with prophylactic antibiotics (Clavamox, PO, ~14 mg/kg) and provided peri-operative analgesia (buprenorphine, SC, 2x daily, 0.05mg/kg) and antibiotics for the first 3 days after surgery. The dorsal skin and superficial muscle layers were dissected to expose the dorsal aspect of the cervical spinal column in isoflurane anaesthetized mice. Ligamentous structures attached to the C4-C7 laminae were carefully removed, and the inferior surface of the laminae were scratched to disrupt the periosteum. In DCM mice, a folded sheet of polyaromatic ether was carefully inserted between the spinal cord and dorsal laminae spanning the C5-C6 segments(Karadimas *et al.*, 2013*b*). For sham animals, all procedures were kept consistent, with the exception that the polyaromatic ether was inserted, then removed to simulate and control for any potential minor trauma associated with insertion. Finally, to check that the spinal cord and spinal roots were not acutely damaged from the procedure, all mice were examined to 24 hours after surgery for signs of neurological deficits. Any indication of motor deficit resulted in the exclusion of the animal from further study.

For Review Only

In Situ Hybridization

In situ hybridization for miR-21 and a positive control, U6 spliceosomal RNA, was performed as previously described(Obernosterer *et al.*, 2007). All solutions for in situ hybridization were ordered RNAse-free or prepared overnight with 0.1% Diethylpyrocarbonate (DEPC, Sigma-Aldrich, St Louis, USA) and autoclaved. Briefly, spinal cord cryosections were dried, post-fixed with 4%PFA, and washed in 1X PBS. Samples were then submerged in an

RNAse-free solution containing triethanolamine, hydrochloric acid and acetic anhydride to acetylate positively charged amino groups and reduce background. Following washing in 1X PBS, tissue sections were permeabilized through treatment with 5 µg/mL RNA grade proteinase K (ThermoFisher Scientific, Waltham, USA) for 5 minutes, and subsequently washed in 1X PBS. Slides were transferred to a humidified hybridization chamber and hybridization solution (50% formamide, 5X SSC buffer, 5X Denhardt's buffer, 500 µg/mL Salmon Sperm DNA, 200 µg/mL Yeast tRNA, 0.02 g/mL blocking reagent (Roche, Basel, Switzerland)) was applied to the tissue before incubation for 4 hours at room temperature. Specific digoxigenin-labeled LNA probes were added to hybridization solution containing 0.25% CHAPS and 0.5% Tween-20, and heat-denatured for 5 minutes at 80˚C before cooling on ice, applying probes to the tissue sections and covering them with Lifterslips™ (ThermoFisher Scientific, Waltham, USA). Slides were then incubated overnight at 53 ˚C to allow probe hybridization. After hybridization, slides were washed in 0.2X Saline-Sodium Citrate and Tris-buffered saline prior to immunohistochemistry using alkaline phosphatase-conjugated anti-digoxigenin sheep polyclonal antibodies (1:500, Roche, Basel, Switzerland). Alkaline phosphatase activity was visualized using the chromogenic nitro-blue tetrazolium and 5-bromo-4-chloro-3'-indolyphosphate substrate (Vector Laboratories, Burlingame, USA).

For Review Only

Primary Microglia Isolation

The cerebellum and meninges were removed from the brain and the remaining brain tissue was minced and dissociated via trituration in cold DMEM. The dissociated cells were then filtered using 40 µm strainers (ThermoFisher Scientific, Waltham, USA), and centrifuged at 300 x g for 10 minutes. Cell pellets were resuspended in DMEM + 10% exosome-depleted fetal bovine serum

593 (FBS, Wisent Bio-Products, St-Bruno, Canada) and seeded in T75 flasks. After 5 days, microglia
594 were isolated from the mixed glial culture by shaking the flasks for 4 hours on an orbital shaker at
595 approximately 70 rpm. The microglia-containing supernatant was collected from the flasks and
596 seeded onto 25mm glass coverslips at a density of 1-2 x10^5.
597

For Review Only

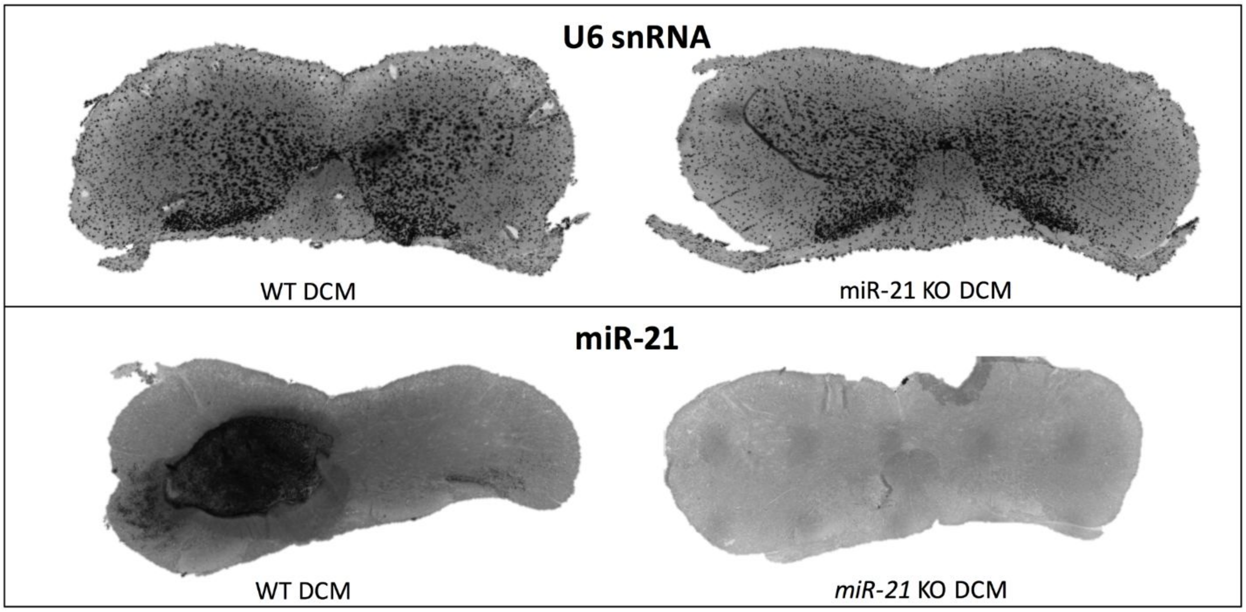

Figure S1. In situ hybridization demonstrates localized overexpression of miR-21 in wild type DCM spinal cords. Chromogenic staining of miR-21 and positive control U6 snRNA was performed using in situ hybridization with digoxigenin-labeled locked nucleic acid probes, and subsequently visualized using alkaline phosphatase-conjugated anti-digoxigenin antibodies and the nitro-blue tetrazolium chloride/5-bromo-4-chloro-3'-indolyphosphate p-toluidine salt substrate system. Spinal cord sections from miR-21 knockout mice were used as negative controls to demonstrate the hybrization specificity.

Table S1. Predicted targets of miR-21 within the IL6/STAT3 gene family. A partial list of STAT3-related factors and receptors with their predicted or validated interaction with miR-21 according to TargetScan, RNA22, and miRdB prediction algorithms and the TarBase validated target database.

	TargetScan	RNA22	miRdB		TargetScan	RNA22	miRdB
IL6/STAT3 Family				Other STAT3-Associated Receptors			
Il6	-	+	-	*Cd4*	-	-	-
Il6ra	+	+	+	*Cd40*	-	-	-
Il6st	+	-	-	*Cd80*	-	-	-
Stat3	+	+	+	*Csf3r*	-	-	-
Lifr	+	-	-	*Cxcr4*	-	-	-
Cntfr	+	+	-	***Fas***	-	-	-
Lif	-	+	-	*Il18r*	-	-	-
Cntf	-	+	-	*Il1r1*	-	-	-
Osm	-	-	-	*Il2ra*	-	-	-
Osmr	-	-	-	***Tnfrsf10b***	-	+	-
Ctf	-	-	-	*Tnfrsf1a*	-	-	-
Clcf1	-	-	-	*Tnfrsf1b*	-	-	-
Il27	-	-	-				
Other STAT3 Activators							
Cxcl12	-	+	-				
Egfr	-	+	-				
Il10	-	-	-				
Il17a	-	-	-				
Il21	-	-	-				

Genes in bold are validated miR-21-5p targets from the TarBase v.8 database(Karagkouni *et al.*, n.d.) Predicted interactions using TargetScan v7.0(Agarwal *et al.*, 2015), RNA22(Miranda *et al.*, 2006), and miRdB(Wong and Wang, 2015) algorithms denoted +

For Review Only

Supplementary References

Agarwal V, Bell GW, Nam J-W, Bartel DP. Predicting effective microRNA target sites in mammalian mRNAs. Elife 2015; 4

Karagkouni D, Paraskevopoulou MD, Chatzopoulos S, Vlachos IS, Tastsoglou S, Kanellos I, et al. DIANA-TarBase v8: a decade-long collection of experimentally supported miRNA–gene interactions [Internet]. Available from: https://doi.org/10.1093/nar/gkx11415

Miranda KC, Huynh T, Tay Y, Ang Y-S, Tam W-L, Thomson AM, et al. A pattern-based method for the identification of MicroRNA binding sites and their corresponding heteroduplexes. Cell 2006; 126: 1203–17.

Obernosterer G, Martinez J, Alenius M. Locked nucleic acid-based in situ detection of microRNAs in mouse tissue sections. Nat Protoc 2007; 2: 1508–14.

Wong N, Wang X. miRDB: an online resource for microRNA target prediction and functional annotations. Nucleic Acids Res 2015; 43: D146-152.

For Review Only

References and Notes:

Agarwal V, Bell GW, Nam J-W, Bartel DP. Predicting effective microRNA target sites in mammalian mRNAs. Elife 2015; 4

Benzel EC, Lancon J, Kesterson L, Hadden T. Cervical laminectomy and dentate ligament section for cervical spondylotic myelopathy. J Spinal Disord 1991; 4: 286–95.

Bergman P, James T, Kular L, Ruhrmann S, Kramarova T, Kvist A, et al. Next-generation sequencing identifies microRNAs that associate with pathogenic autoimmune neuroinflammation in rats. J Immunol 2013; 190: 4066–75.

Bhalala OG, Pan L, Sahni V, McGuire TL, Gruner K, Tourtellotte WG, et al. microRNA-21 Regulates Astrocytic Response Following Spinal Cord Injury. J Neurosci 2012; 32: 17935–47.

Bourquin AF, Suveges M, Pertin M, Gilliard N, Sardy S, Davison AC, et al. Assessment and analysis of mechanical allodynia-like behavior induced by spared nerve injury (SNI) in the mouse. Pain 2006; 122: 14-e14.

Breig A, Turnbull I, Hassler O. Effects of mechanical stresses on the spinal cord in cervical spondylosis. A study on fresh cadaver material. J Neurosurg 1966; 25: 45–56.

Broggini T, Schnell L, Ghoochani A, Mateos JM, Buchfelder M, Wiendieck K, et al. Plasticity Related Gene 3 (PRG3) overcomes myelin-associated growth inhibition and promotes functional recovery after spinal cord injury. Aging (Albany NY) 2016; 8: 2463–83.

Cui G-H, Wu J, Mou F-F, Xie W-H, Wang F-B, Wang Q-L, et al. Exosomes derived from hypoxia-preconditioned mesenchymal stromal cells ameliorate cognitive decline by rescuing synaptic dysfunction and regulating inflammatory responses in APP/PS1 mice. FASEB J 2017

DeLeo JA, Colburn RW, Nichols M, Malhotra A. Interleukin-6-mediated hyperalgesia/allodynia and increased spinal IL-6 expression in a rat mononeuropathy model. J Interferon Cytokine Res 1996; 16: 695–700.

Dominguez E, Mauborgne A, Mallet J, Desclaux M, Pohl M. SOCS3-mediated blockade of JAK/STAT3 signaling pathway reveals its major contribution to spinal cord neuroinflammation and mechanical allodynia after peripheral nerve injury. J Neurosci 2010; 30: 5754–66.

Dominguez E, Rivat C, Pommier B, Mauborgne A, Pohl M. JAK/STAT3 pathway is activated in spinal cord microglia after peripheral nerve injury and contributes to neuropathic pain development in rat. J Neurochem 2008; 107: 50–60.

Fehlings MG, Ibrahim A, Tetreault L, Albanese V, Alvarado M, Arnold P, et al. A global perspective on the outcomes of surgical decompression in patients with cervical spondylotic myelopathy: results from the prospective multicenter AOSpine international study on 479 patients. Spine 2015; 40: 1322–8.

Fernando MR, Reyes JL, Iannuzzi J, Leung G, McKay DM. The Pro-Inflammatory Cytokine, Interleukin-6, Enhances the Polarization of Alternatively Activated Macrophages. PLOS ONE 2014; 9: e94188.

Fielding CA, McLoughlin RM, McLeod L, Colmont CS, Najdovska M, Grail D, et al. IL-6 regulates neutrophil trafficking during acute inflammation via STAT3. J Immunol 2008; 181: 2189–95.

Gaudet AD, Fonken LK, Watkins LR, Nelson RJ, Popovich PG. MicroRNAs: Roles in Regulating Neuroinflammation. Neuroscientist 2017: 1073858417721150.

Ge X-T, Lei P, Wang H-C, Zhang A-L, Han Z-L, Chen X, et al. miR-21 improves the neurological outcome after traumatic brain injury in rats. Sci Rep 2014; 4: 6718.

For Review Only

Harrison EB, Hochfelder CG, Lamberty BG, Meays BM, Morsey BM, Kelso ML, et al. Traumatic brain injury increases levels of miR-21 in extracellular vesicles: implications for neuroinflammation. FEBS Open Bio 2016; 6: 835–46.

Hirai T, Uchida K, Nakajima H, Guerrero AR, Takeura N, Watanabe S, et al. The Prevalence and Phenotype of Activated Microglia/Macrophages within the Spinal Cord of the Hyperostotic Mouse (twy/twy) Changes in Response to Chronic Progressive Spinal Cord Compression: Implications for Human Cervical Compressive Myelopathy [Internet]. PLoS One 2013; 8[cited 2016 May 5] Available from: http://www.ncbi.nlm.nih.gov/pmc/articles/PMC3663759/

Hori N, Narita M, Yamashita A, Horiuchi H, Hamada Y, Kondo T, et al. Changes in the expression of IL-6-Mediated MicroRNAs in the dorsal root ganglion under neuropathic pain in mice. Synapse 2016; 70: 317–24.

Hu J-Z, Huang J-H, Zeng L, Wang G, Cao M, Lu H-B. Anti-apoptotic effect of microRNA-21 after contusion spinal cord injury in rats. J Neurotrauma 2013; 30: 1349–60.

Jia Z, Lian W, Shi H, Cao C, Han S, Wang K, et al. Ischemic Postconditioning Protects Against Intestinal Ischemia/Reperfusion Injury via the HIF-1α/miR-21 Axis. Sci Rep 2017; 7: 16190.

Kameda T, Kaneuchi Y, Sekiguchi M, Konno S. Measurement of mechanical withdrawal thresholds and gait analysis using the CatWalk method in a nucleus pulposus-applied rodent model. Journal of Experimental Orthopaedics 2017; 4: 31.

Karadimas SK, Erwin WM, Ely CG, Dettori JR, Fehlings MG. Pathophysiology and natural history of cervical spondylotic myelopathy. Spine 2013; 38: S21-36.

Karadimas SK, Gatzounis G, Fehlings MG. Pathobiology of cervical spondylotic myelopathy. Eur Spine J 2015; 24: 132–8.

Karadimas SK, Laliberte AM, Tetreault L, Chung YS, Arnold P, Foltz WD, et al. Riluzole blocks perioperative ischemia-reperfusion injury and enhances postdecompression outcomes in cervical spondylotic myelopathy. Science Translational Medicine 2015; 7: 316ra194-316ra194.

Karadimas SK, Moon ES, Yu W-R, Satkunendrarajah K, Kallitsis JK, Gatzounis G, et al. A novel experimental model of cervical spondylotic myelopathy (CSM) to facilitate translational research. Neurobiology of Disease 2013; 54: 43–58.

Karagkouni D, Paraskevopoulou MD, Chatzopoulos S, Vlachos IS, Tastsoglou S, Kanellos I, et al. DIANA-TarBase v8: a decade-long collection of experimentally supported miRNA–gene interactions [Internet]. Available from: https://doi.org/10.1093/nar/gkx1141

Karl F, Grießhammer A, Üçeyler N, Sommer C. Differential Impact of miR-21 on Pain and Associated Affective and Cognitive Behavior after Spared Nerve Injury in B7-H1 ko Mouse. Front Mol Neurosci 2017; 10: 219.

Kovalova I, Kerkovsky M, Kadanka Z, Kadanka Z, Nemec M, Jurova B, et al. Prevalence and Imaging Characteristics of Nonmyelopathic and Myelopathic Spondylotic Cervical Cord Compression. Spine 2016; 41: 1908–16.

Lam D, Lively S, Schlichter LC. Responses of rat and mouse primary microglia to pro- and anti-inflammatory stimuli: molecular profiles, K+ channels and migration [Internet]. J Neuroinflammation 2017; 14[cited 2017 Nov 28] Available from: https://www.ncbi.nlm.nih.gov/pmc/articles/PMC5567442/

Langeslag M, Constantin CE, Andratsch M, Quarta S, Mair N, Kress M. Oncostatin M induces heat hypersensitivity by gp130-dependent sensitization of TRPV1 in sensory neurons. Mol Pain 2011; 7: 102.

For Review Only

705 Leinders M, Üçeyler N, Thomann A, Sommer C. Aberrant microRNA expression in patients with painful peripheral neuropathies. J Neurol Sci 2017; 380: 242–9.

707 Li FN, Li ZH, Huang X, Yu SZ, Zhang F, Chen Z, et al. The treatment of mild cervical spondylotic myelopathy with increased signal intensity on T2-weighted magnetic resonance imaging. Spinal Cord 2014; 52: 348–53.

709 Liu N-K, Wang X-F, Lu Q-B, Xu X-M. Altered microRNA expression following traumatic spinal cord injury. Exp Neurol 2009; 219: 424–9.

711 Liu X, Tian Y, Lu N, Gin T, Cheng CHK, Chan MTV. Stat3 Inhibition Attenuates Mechanical Allodynia through Transcriptional Regulation of Chemokine Expression in Spinal Astrocytes [Internet]. PLoS One 2013; 8[cited 2017 Nov 27] Available from: https://www.ncbi.nlm.nih.gov/pmc/articles/PMC3789727/

714 Malsch P, Andratsch M, Vogl C, Link AS, Alzheimer C, Brierley SM, et al. Deletion of interleukin-6 signal transducer gp130 in small sensory neurons attenuates mechanonociception and down-regulates TRPA1 expression. J Neurosci 2014; 34: 9845–56.

717 McLoughlin RM, Jenkins BJ, Grail D, Williams AS, Fielding CA, Parker CR, et al. IL-6 trans-signaling via STAT3 directs T cell infiltration in acute inflammation. Proc Natl Acad Sci USA 2005; 102: 9589–94.

719 Miranda KC, Huynh T, Tay Y, Ang Y-S, Tam W-L, Thomson AM, et al. A pattern-based method for the identification of MicroRNA binding sites and their corresponding heteroduplexes. Cell 2006; 126: 1203–17.

721 Miyagi M, Ishikawa T, Kamoda H, Suzuki M, Sakuma Y, Orita S, et al. Assessment of pain behavior in a rat model of intervertebral disc injury using the CatWalk gait analysis system. Spine 2013; 38: 1459–65.

723 Molet J, Mauborgne A, Diallo M, Armand V, Geny D, Villanueva L, et al. Microglial Janus kinase/signal transduction and activator of transcription 3 pathway activity directly impacts astrocyte and spinal neuron characteristics. J Neurochem 2016; 136: 133–47.

726 Moon ES, Karadimas SK, Yu W-R, Austin JW, Fehlings MG. Riluzole attenuates neuropathic pain and enhances functional recovery in a rodent model of cervical spondylotic myelopathy. Neurobiology of Disease 2014; 62: 394–406.

729 Murugaiyan G, da Cunha AP, Ajay AK, Joller N, Garo LP, Kumaradevan S, et al. MicroRNA-21 promotes Th17 differentiation and mediates experimental autoimmune encephalomyelitis. J Clin Invest 2015; 125: 1069–80.

731 Nagashima H, Morio Y, Yamane K, Nanjo Y, Teshima R. Tumor necrosis factor-α, interleukin-1β, and interleukin-6 in the cerebrospinal fluid of patients with cervical myelopathy and lumbar radiculopathy. Eur Spine J 2009; 18: 1946–50.

734 Nouri A, Tetreault L, Singh A, Karadimas SK, Fehlings MG. Degenerative Cervical Myelopathy: Epidemiology, Genetics, and Pathogenesis. Spine 2015; 40: E675–93.

736 Nurick S. The pathogenesis of the spinal cord disorder associated with cervical spondylosis. Brain 1972; 95: 87–100.

737 Obernosterer G, Martinez J, Alenius M. Locked nucleic acid-based in situ detection of microRNAs in mouse tissue sections. Nat Protoc 2007; 2: 1508–14.

739 Oshima Y, Seichi A, Takeshita K, Chikuda H, Ono T, Baba S, et al. Natural course and prognostic factors in patients with mild cervical spondylotic myelopathy with increased signal intensity on T2-weighted magnetic resonance imaging. Spine 2012; 37: 1909–13.

For Review Only

Quarta S, Vogl C, Constantin CE, Üçeyler N, Sommer C, Kress M. Genetic evidence for an essential role of neuronally expressed IL-6 signal transducer gp130 in the induction and maintenance of experimentally induced mechanical hypersensitivity in vivo and in vitro. Mol Pain 2011; 7: 73.

Romano M, Sironi M, Toniatti C, Polentarutti N, Fruscella P, Ghezzi P, et al. Role of IL-6 and Its Soluble Receptor in Induction of Chemokines and Leukocyte Recruitment. Immunity 1997; 6: 315–25.

Takeura N, Nakajima H, Watanabe S, Honjoh K, Takahashi A, Matsumine A. Role of macrophages and activated microglia in neuropathic pain associated with chronic progressive spinal cord compression. Sci Rep 2019; 9: 15656.

Tetreault L, Kopjar B, Nouri A, Arnold P, Barbagallo G, Bartels R, et al. The modified Japanese Orthopaedic Association scale: establishing criteria for mild, moderate and severe impairment in patients with degenerative cervical myelopathy. Eur Spine J 2017; 26: 78–84.

Tsuda M, Kohro Y, Yano T, Tsujikawa T, Kitano J, Tozaki-Saitoh H, et al. JAK-STAT3 pathway regulates spinal astrocyte proliferation and neuropathic pain maintenance in rats. Brain 2011; 134: 1127–39.

Vidal P, Karadimas SK, Ulndreaj A, Laliberte AM, Tetreault L, Forner S, et al. Delayed decompression exacerbates ischemia-reperfusion injury in cervical compressive myelopathy. JCI Insight 2017; 2

Vidal PM, Lemmens E, Avila A, Vangansewinkel T, Chalaris A, Rose-John S, et al. ADAM17 is a survival factor for microglial cells in vitro and in vivo after spinal cord injury in mice. Cell Death Dis 2013; 4: e954.

Vissers KC, De Jongh RF, Hoffmann VL, Meert TF. Exogenous interleukin-6 increases cold allodynia in rats with a mononeuropathy. Cytokine 2005; 30: 154–9.

Wang Z, Brandt S, Medeiros A, Wang S, Wu H, Dent A, et al. MicroRNA 21 Is a Homeostatic Regulator of Macrophage Polarization and Prevents Prostaglandin E 2 -Mediated M2 Generation. PLOS ONE 2015; 10: e0115855.

Wong N, Wang X. miRDB: an online resource for microRNA target prediction and functional annotations. Nucleic Acids Res 2015; 43: D146-152.

Yasukawa H, Ohishi M, Mori H, Murakami M, Chinen T, Aki D, et al. IL-6 induces an anti-inflammatory response in the absence of SOCS3 in macrophages. Nat Immunol 2003; 4: 551–6.

Yelamanchili SV, Lamberty BG, Rennard DA, Morsey BM, Hochfelder CG, Meays BM, et al. MiR-21 in Extracellular Vesicles Leads to Neurotoxicity via TLR7 Signaling in SIV Neurological Disease. PLOS Pathogens 2015; 11: e1005032.

Yu WR, Liu T, Kiehl T-R, Fehlings MG. Human neuropathological and animal model evidence supporting a role for Fas-mediated apoptosis and inflammation in cervical spondylotic myelopathy. Brain 2011; 134: 1277–92.

Yunta M, Nieto-Díaz M, Esteban FJ, Caballero-López M, Navarro-Ruíz R, Reigada D, et al. MicroRNA dysregulation in the spinal cord following traumatic injury. PLoS ONE 2012; 7: e34534.

Zhong L, Xiao W, Wang F, Liu J, Zhi L-J. miR-21-5p inhibits neuropathic pain development via directly targeting C-C motif ligand 1 and tissue inhibitor of metalloproteinase-3. J Cell Biochem 2019; 120: 16614–23.

Ziu M, Fletcher L, Rana S, Jimenez DF, Digicaylioglu M. Temporal differences in microRNA expression patterns in astrocytes and neurons after ischemic injury. PLoS ONE 2011; 6: e14724.

For Review Only

Figures

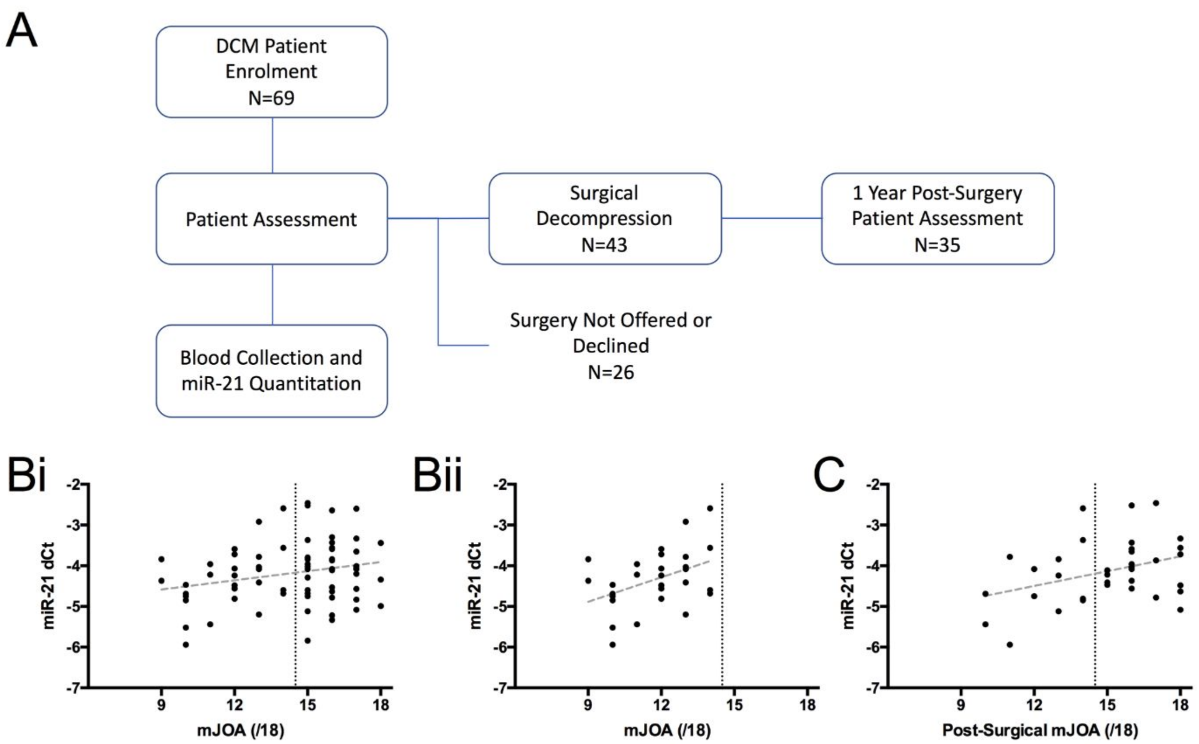

Figure 1. miR-21 expression correlates with severity of neurological deficits in human DCM patients at baseline and following surgical treatment. A) A schematic showing the experimental design and sample size for the patient cohort. Bi) Normalized miR-21 expression, denoted as dCt values (where each -1 dCt represents a doubling of expression), was modestly correlated to DCM severity at the time of initial assessment (Pearson Correlation test, r=0.248, p=0.049). Bii) This correlation was stronger in the subset of patients with moderate to severe deficits with an mJOA<15(Tetreault *et al.*, 2017) (r=0.424, p=0.025, n=28). C) Initial miR-21 expression was also significantly correlated to DCM patient outcome, assessed 1 year after surgical decompression (r=0.368, p=0.030, n=35).

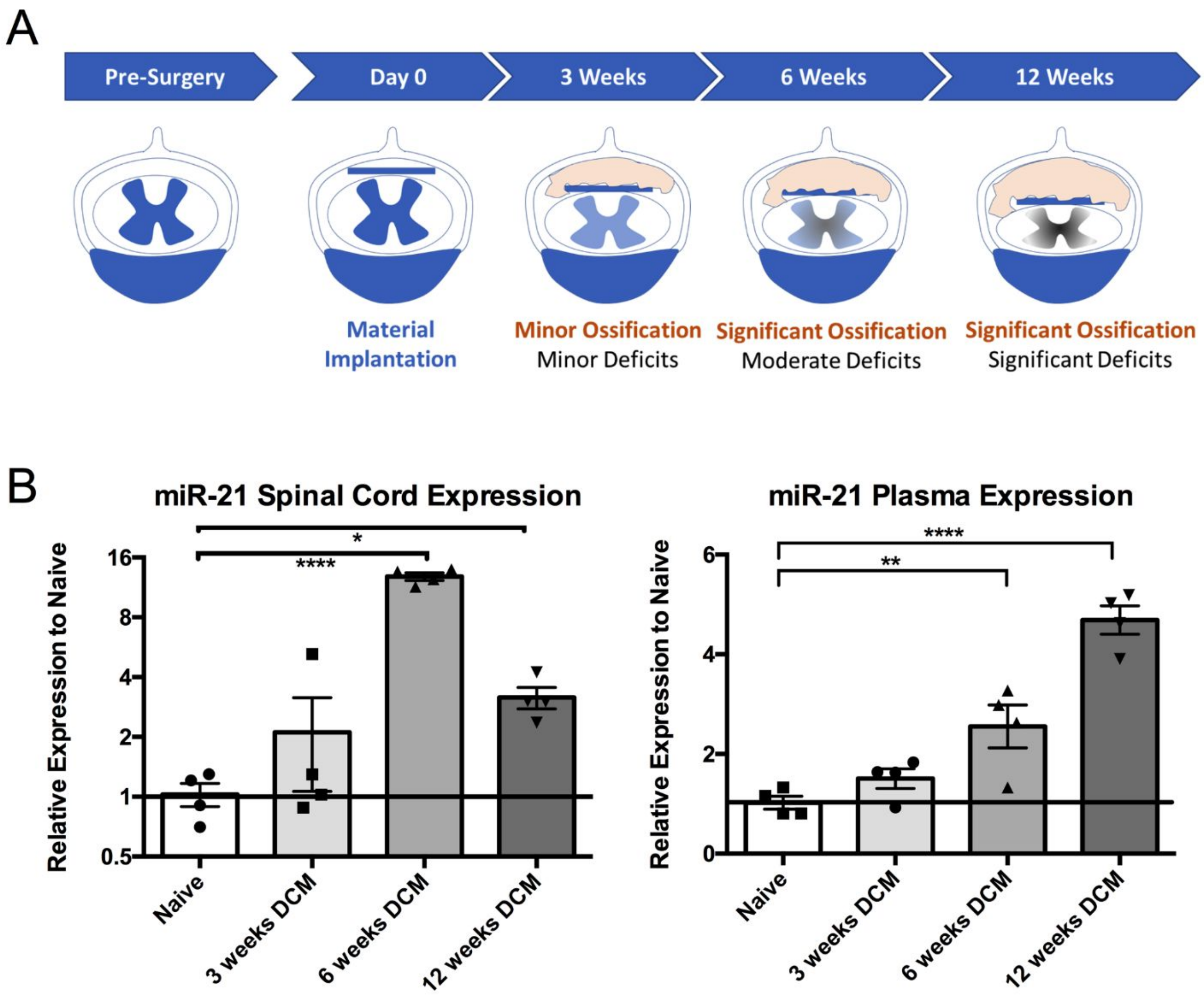

Figure 2. miR-21 expression is increased in a mouse model of degenerative cervical myelopathy. A) A schematic illustrating the timeline of the progressive spinal cord compression induced in the mouse DCM model B) miR-21 expression results from real-time quantitative PCR demonstrate an increase in spinal cord expression at 6 and 12 weeks (ANOVA, Dunnett's post hoc, q=8.002 and 3.54, respectively, df=12), and an increase in plasma at 6 and 12 weeks (ANOVA, Dunnett's post hoc, q=4.272 and 7.453, respectively, df=12). Spinal cord miR-21 was normalized to U6 snRNA, and plasma miR-21 to miR-16. n=4 mice. Error bars = ± SEM, *(p<0.05), **(p<0.01), ****(p<0.0001).

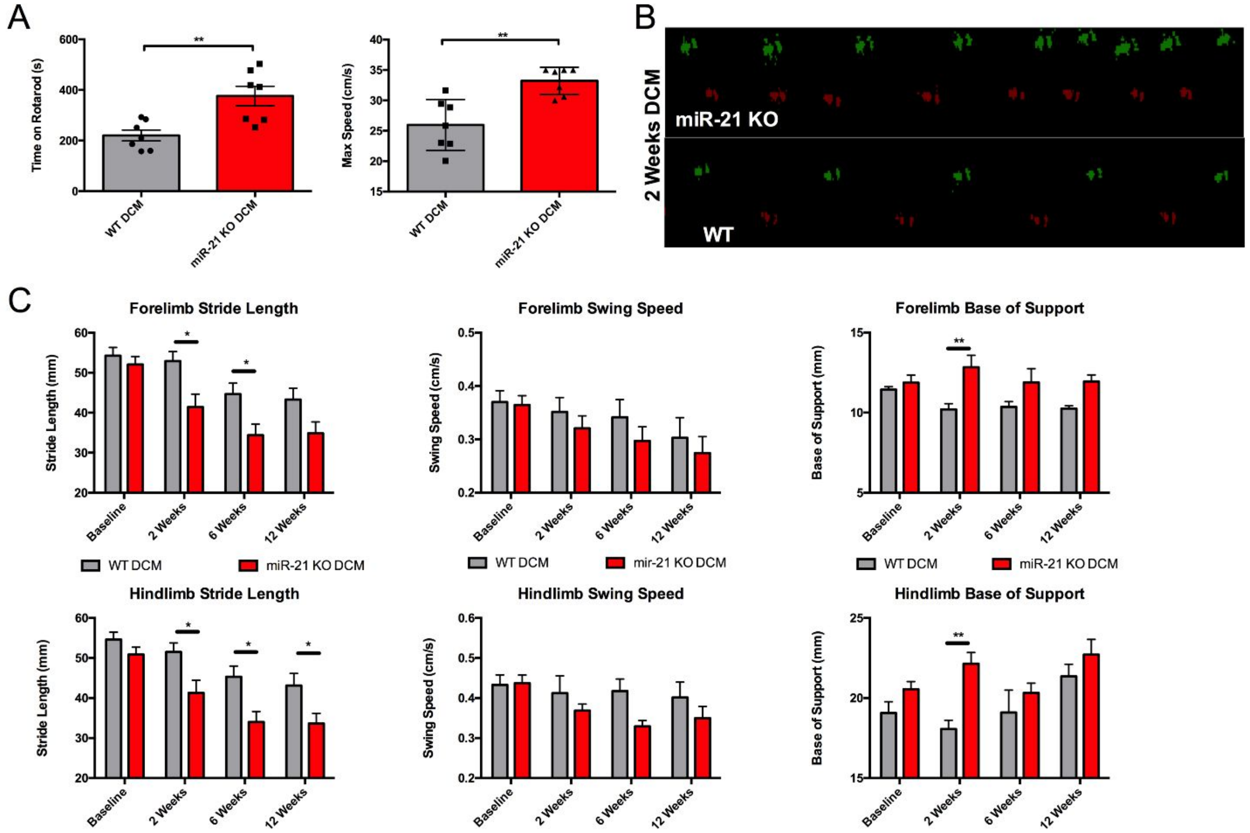

Figure 3. Deletion of miR-21 preserves locomotor function on the rotarod test, but generates gait abnormalities during spontaneous movement in mice with degenerative cervical myelopathy (DCM). A) miR-21 knockout animals with DCM for 12 weeks were able to maintain balance for significantly longer than wild type DCM animals, and at significantly higher speed while the rotarod accelerated from 3.5 to 35 rpm (t-test, $p=0.004$, $t=3.547$, and $p=0.0017$, $t= 4.031$, respectively, df=12). B) Representative hindlimb traces from 2 week miR-21 knockout and wild type DCM mice. miR-21 KO DCM mice demonstrate a shorter stride length at an early time point that is typically pre-symptomatic in WT animals. C) Repeated measures ANOVA of Catwalk gait results identified significant decreases in forelimb stride length at 2 weeks (Sidak's post-hoc, $t=3.083$, DF=48) and 6 weeks (Sidak's post-hoc, $t=2.747$, DF=48), and decreases in hindlimb stride length at 2 weeks (Sidak's post-hoc, $t=2.846$, DF=48), 6 weeks (Sidak's post-hoc, $t=3.144$, DF=48), and 12 weeks (Sidak's post-hoc, $t=2.641$, DF=48) in miR-21 KO DCM mice compared to WT DCM mice. Animal miR-21 status was identified as a significant factor for hindlimb swing speed (repeated measures ANOVA, $F=7.658$, DF=1,12, $p=0.017$), but multiple comparisons testing did not identify specific differences at any of the time points tested. Forepaw and hindpaw base of support were both significantly increased in miR-21 KO DCM mice at 2 weeks (Sidak's post-hoc, $t=3.722$, DF=48, and $t=3.539$, DF=48, respectively). n=7 mice. Error bars = ± SEM, *($p<0.05$), **($p<0.01$), ***($p<0.001$), ****($p<0.0001$).

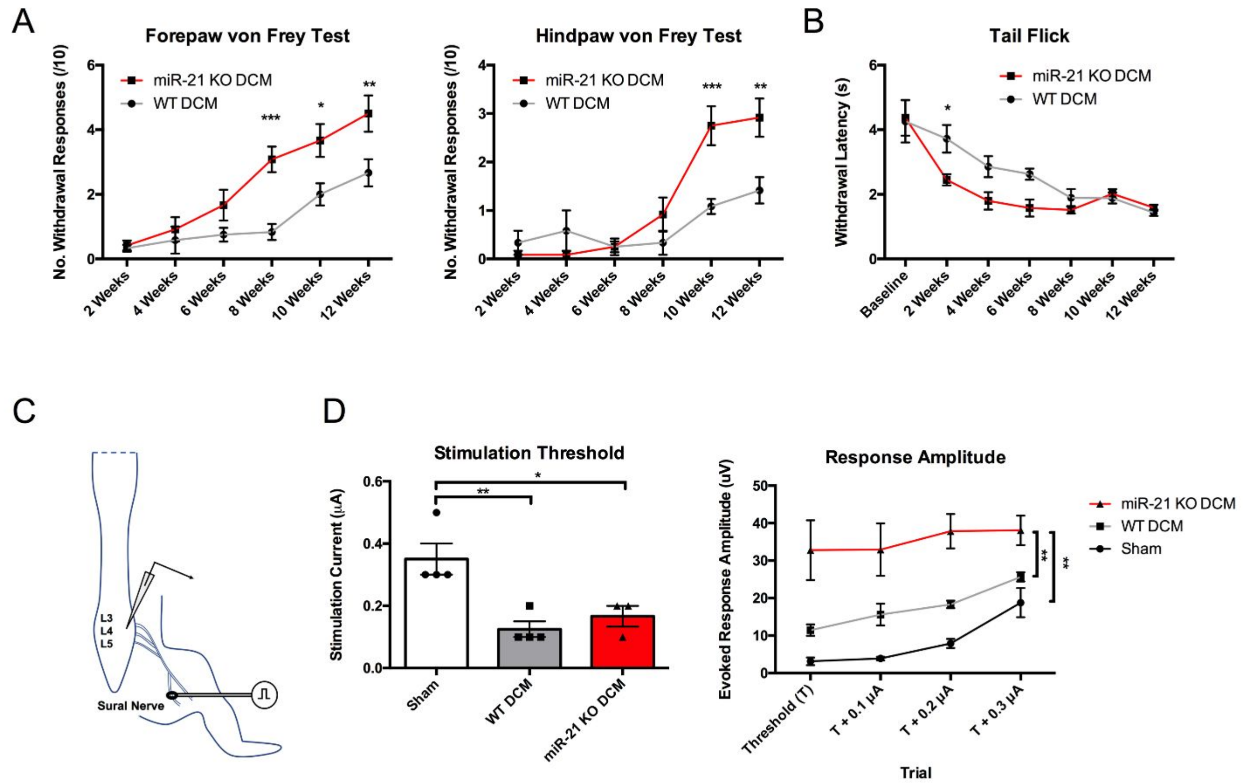

Figure 4. Sensory assessment using the von Frey and tail flick tests identify evidence of increased mechanical allodynia and thermal hyperalgesia in miR-21 KO mice. A) The frequency of paw withdrawal responses to touch with a calibrated 0.4g von Frey monofilament were significantly higher in forepaws of miR-21 KO degenerative cervical myelopathy (DCM) mice, specifically at 8 weeks, 10 weeks and 12 weeks (Repeated Measures ANOVA, Sidak's post-hoc, t=4.212, t=3.12, and t=3.432, respectively, df=60). Hindpaw von Frey assessment also found significantly higher withdrawal frequency in miR-21 KO DCM mice at weeks 10 and 12 (Repeated Measures ANOVA, Sidak's post-hoc, t=4.323 and t=3.891, respectively, DF=60). B) The latency of withdrawal from a mildly noxious heat stimulus was significantly reduced in miR-21 KO DCM mice relative to wild type DCM at 2 weeks (Repeated Measures ANOVA, Sidak's post-hoc, t=2.862, DF=70), suggesting increased thermal hyperalgesia in the early stages of disease progression. n=6 mice. C) Schematic of dorsal horn field potential recordings during sural nerve stimulation. D) The minimum stimulation threshold and evoked response amplitude from lumbar dorsal horn field recordings. The stimulation threshold from sural nerve stimulation was significantly decreased in both wild type DCM and miR-21 KO DCM mice (ANOVA, Tukey's post-hoc, p=0.0067, q=6.056 and p=0.029, q=4.569, respectively, DF=8). However, the evoked response amplitude of miR-21 KO mice (n=3) was significantly higher than both WT DCM (n=4) and Sham (n=4) groups (Repeated Measures ANOVA, Tukey's post-hoc, p=0.008, and p=0.001, respectively, DF=8). Error bars = ± SEM, *(p<0.05), **(p<0.01), ***(p<0.001), ****(p<0.0001).

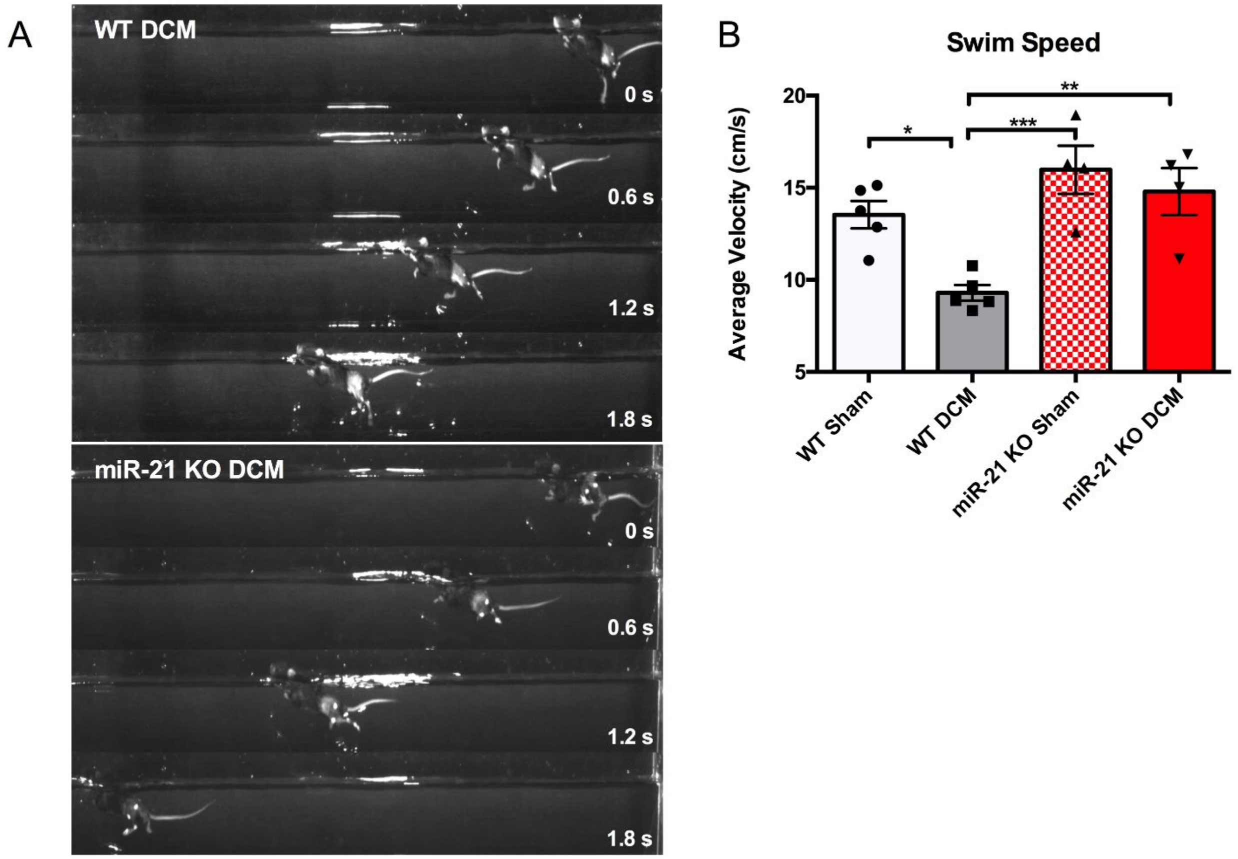

Figure 5. miR-21 knockout mice with degenerative cervical myelopathy (DCM) swim significantly faster in a forced swim test than wild type DCM mice. A) A representative montage of wild type and miR-21 KO DCM animals swimming over 1.8 seconds. B) 12-week miR-21 KO DCM mice were not significantly different from wild-type and knockout sham animals, but were significantly faster swimmers than wild-type DCM mice (ANOVA, Tukey's post-hoc, q=5.873, DF=14). n=5 mice for wild-type groups, n=4 mice for miR-21 KO groups. Error bars = ± SEM, *(p<0.05), **(p<0.01), ***(p<0.001), ****(p<0.0001).

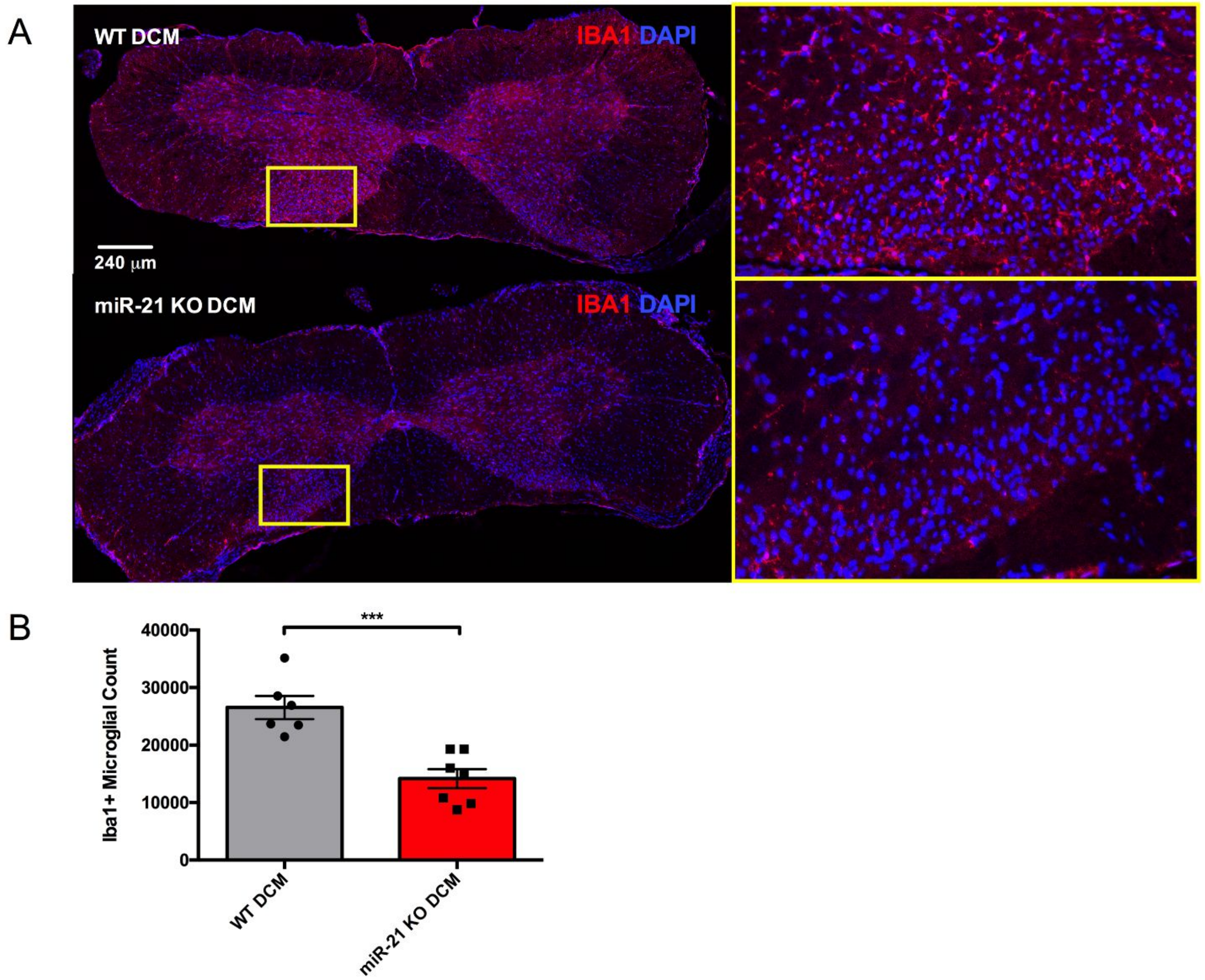

Figure 6. Iba1 immunohistochemistry and stereological counting identifies significant reduction of Iba1+ microglia in miR-21 KO degenerative cervical myelopathy (DCM) mice relative to wild-type DCM mice. A) Representative images of the compressed regions of wild-type and miR-21 KO DCM spinal cords. High magnification insets highlight the differences in Iba1 staining in the dorsal grey matter. B) A bar graph illustrating the results of stereological counts of Iba1-positive/DAPI-positive microglia throughout 1.92 mm of the compressed area of the cervical spinal cord (t-test, p=0.0006, t=4.785, df=11). WT DCM (n=6 mice), miR-21 KO DCM (n=7 mice). Error bars = ± SEM, *(p<0.05), **(p<0.01), ***(p<0.001), ****(p<0.0001).

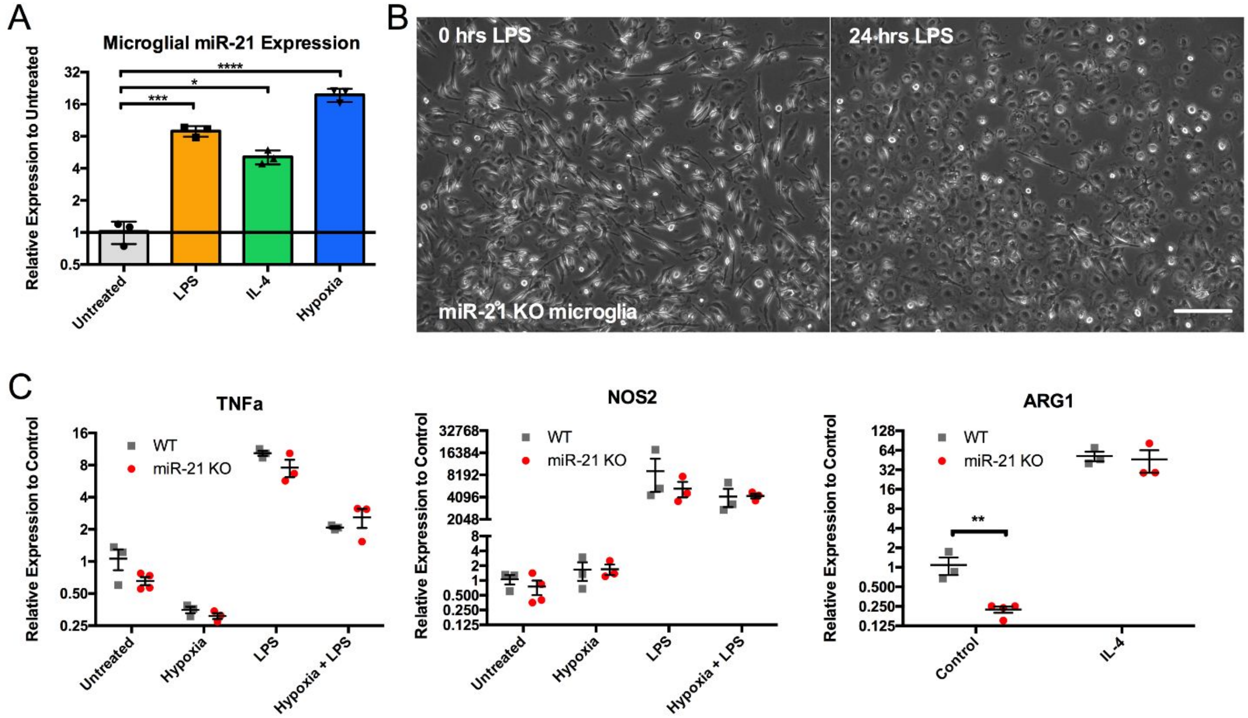

Figure 7. Primary microglia express miR-21 following pro- and anti-inflammatory treatments. A) miR-21 expression was significantly increased relative to controls 24 hours after treatment with either 10 ng/mL LPS (ANOVA, Dunnett's post-hoc, $p=0.0006$, $q=6.293$, DF=8) , 20 ng/mL IL-4 (ANOVA, Dunnett's post-hoc, $p=0.0289$, $q=3.253$, DF=8), or 24-hour incubation at 1% O_2 (ANOVA, Dunnett's post-hoc, $p<0.0001$, $q=14.73$, DF=8). Untreated microglial cultures (n=3), LPS-treated (n=3), IL-4-treated (n=3), Hypoxia-treated (n=3). B) Representative image showing miR-21 KO microglia retracting processes and adopting the classical amoeboid morphology by 12 hours after the addition of LPS. [Scale bar = 160 µm] C) Typical markers of M1 and M2 polarized macrophages are not differentially expressed between wild type (WT) and miR-21 knockout (miR-21 KO) microglia following the addition of pro- and anti-inflammatory stimuli. MiR-21 genotype was not a significant factor for TNFa (two-way ANOVA, $p= 0.0879$, $F=3.279$, $DF_{(n,d)}=1,17$) or NOS2 (two-way ANOVA, $p= 0.2920$, $F=1.182$, $DF_{(n,d)}=1,17$). There was a statistically significant effect of genotype on ARG1 (two-way ANOVA, $p=0.0046$, $F=13.98$, $DF_{(n,d)}=1,9$), but multiple comparisons testing found this difference only for untreated microglia (Sidak's post-hoc, $p=0.0020$, $t=4.787$, DF=9), and not those treated with IL-4 (Sidak's post-hoc, $p=0.7862$, $t=0.6408$, DF=9). WT untreated microglia (n=3), WT hypoxia (n=3), WT LPS (n=3), WT LPS + hypoxia (n=3), WT IL-4 (n=3), miR-21 KO untreated (n=4), miR-21 KO hypoxia (n=3), miR-21 KO LPS (n=3), miR-21 KO LPS + hypoxia (n=3), miR-21 KO IL-4 (n=3). Error bars = ± SEM, *($p<0.05$), **($p<0.01$), ***($p<0.001$), ****($p<0.0001$).

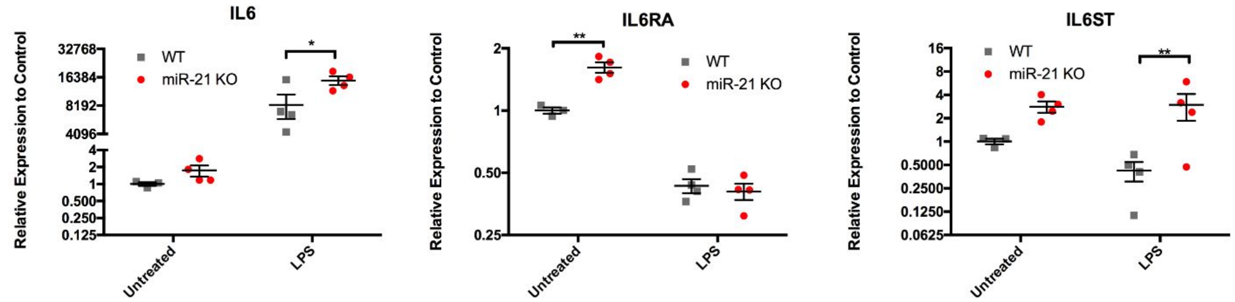

Figure 8. Deletion of miR-21 results in significantly higher expression of Il6, Il6ra, and Il6st in primary microglia. MiR-21 genotype was a statistically significant factor for Il6 (two-way ANOVA, p=0.0112, F=9.263, $DF_{(n,d)}$=1,11), Il6ra (two-way ANOVA, p=0.0190, F=7.538, $DF_{(n,d)}$=1,11), and Il6st (two-way ANOVA, p=0.0034, F=13.83, $DF_{(n,d)}$=1,11). Specifically, expression of Il6 and Il6st was significantly increased in miR-21 knockout microglia compared to wild type microglia following the addition of 10 ng/mL LPS (Sidak's post-hoc, p=0.0469, t=2.623; p=0.0095, t=3.257, respectively, DF=11). Il6ra was not different between genotypes after LPS addition, but was higher in miR-21 KO microglia in the untreated group (Sidak's post-hoc, p=0.0023, t=4.357, DF=11). WT untreated microglial cultures (n=3), WT LPS (n=4), miR-21 KO untreated (n=4), miR-21 KO LPS (n=4). Error bars = ± SEM, *(p<0.05), **(p<0.01), ***(p<0.001), ****(p<0.0001).

For Review Only
