# Supplementary material for: Mir21 modulates inflammation and sensorimotor deficits in cervical myelopathy: data from humans and animal models
Source: Brain Commun. 2021 Jan 21;3(1):fcaa234. doi: 10.1093/braincomms/fcaa234 (PMC7878254; doi:10.1093/braincomms/fcaa234)
Supplement: fcaa234_Supplementary_Data [file Original Submission Authors Response.pdf › Original Submission Authors Response.pdf]

Response to Reviewers Comments to Author

Reviewer: 1

Comments to the Author
Laliberte and colleagues have investigated the role that miR-21 plays in the progression of degenerative cervical myelopathy through using human plasma samples, a knock-out mouse line, and primary mouse microglia lines. As a whole, the paper provides convincing evidence that miR-21 is a critical target for investigation in DCM. As such, I am pleased to say that I recommend this manuscript to be accepted after addressing the following comments below:

As is standard since 2015, can the authors please put whether they are referring to the -3p or -5p arm of the miRNA throughout the manuscript as doing so in the abbreviations would not be acceptable. Otherwise, I could take this to mean that the precursor is being referred to and that both -3p and -5p are involved.

We thank the reviewer for identifying this oversight. We have updated the manuscript to indicate the arm of the miRNA where appropriate (-5p). In cases where the text is referring to the miR-21 gene (encoding both -3p and -5p arms), it has been left as is.

Introduction:
Are there any other miRNA associated with DCM? You only mention miR-21 and I am wondering whether there are others that have been linked previously, and if so, why these were investigated during the human plasma study. If not though, it would still be useful to have this stated in the introduction.

At the time of submission, we were unable to find any other published studies demonstrating an association between miRNA and DCM. The introduction has been revised to state this point (pg 4, ln 63-64).

Methods:
L161: What was the volume of plasma that was utilised in extracting the samples? Even if it was the default amount stated in the instructions, it is good to state these for clarity especially as the starting volume can be up to 900ul.

We strongly agree that the volume of plasma input is critical for this methodology – as high input volumes can lead to higher concentrations of contaminants and/or PCR inhibitors. As such, we used the recommended volumes of 200 uL plasma for human samples and 50 uL plasma (diluted with RNAse-free water to 200 uL) for mouse samples. These details have been added to the methods.

L165: Consequently, how much RNA was loaded into the cDNA reactons? In terms of serum, was this the recommended starting volume amount?

In each case, the recommended amount of RNA was used according to the kit manufacturer's instructions. For plasma samples, the amount of input small RNA is not reliably quantifiable using standard spectrophotometric assays, so the input amounts are generally based on the initial volume of plasma. For human samples, each 20 uL RT reaction used a volume of RNA corresponding to 16 µL of the original plasma. Essentially, 200 uL of plasma was processed to 50 uL of RNA eluate (4:1), then 4

uL of this RNA was used in a cDNA synthesis reaction. Due to the higher concentration of PCR inhibitors in mouse plasma/serum samples, the recommended input amount was lower – corresponding to 4 µL of mouse plasma. For microRNA quantitation from spinal cord samples, the input amount was 20 ng RNA per 20 µL RT reaction. For mRNA quantitation from spinal cord samples, the input amount was approximately 100 ng RNA per 20 µL RT reaction. A table listing these amounts is now provided in the supplementary information (Table S1).

L171: There is no such thing as real-time quantitative PCR – real time and quantitative describe the same thing – RT should be referring to the fact you did a reverse transcription step.

We included 'real-time' to distinguish from older quantitative PCR methodologies that compared changes in fluorescence at specific amplification cycles (such as semi-quantitative PCR). However, given the broad adoption of qPCR to signify real-time PCR, the reviewer's point is well taken, and we have modified manuscript accordingly.

L176: How were your normalisers chosen? Normalisers are extremely difficult to select for in plasma data without having undertaken screening experiments like RNA-seq as miRNA can be highly variable between samples. Can you please demonstrate that they were not changed between your samples and were stable across your samples through providing average ± SD of the raw Cq values.

The selection of normalizers was informed by the literature and by subsequent examination of expression stability in experimental samples. As requested, we have provided the raw Cq ± SD for miR-423-5p and 4 other candidate normalizers tested during the preliminary stages of this study. These preliminary data have now been included in the supplementary information (Table S2) of the revised manuscript. Of these candidates, miR-423-5p performed the best in a mixed sample of healthy control (n=10), mild DCM (n=20), and moderate to severe DCM (n=10) samples.

L177: For all mRNA TaqMan probes and primer sets, please provide the catalog numbers as there are multiple primer sets for each gene, so these must be provided at least in a supplementary table.

A table has been added to the supplementary information with all the primer/probe information relevant to the study (Table S3).

L202: For the Catwalk Gait analysis, how many runs were not analysed because they were interrupted? I am curious whether in addition to their disrupted gait whether they also had increased stop/start movements.

The reviewer has correctly identified a feature of the DCM mouse locomotor phenotype – an increased tendency for stops and starts. In this case, we did not record the number of runs that were not analyzed because of a premature stop. While it might be possible to re-analyse the catwalk files to find the number of runs with premature stops, we suspect that it would not be fruitful, since it would be extremely difficult to determine from the recordings (which only show the paws of the animals) whether the mice stopped as a result of disrupted gait or due to distraction or exploratory behaviour.

Results:
L300: I am not sure that a correlation between post-surgical mJOA and miR-21 dCt show anything. If they already have a low score, how much can this change post-surgery? I think a more useful analysis

would be to look at the change in score before and after surgery and compare, as if there is an correlation between pre-surgery miR-21 dCt and post-surgery mJOA, then it should show a correlation.

Thank you for this suggestion. The range of outcomes, even for individuals with low pre-surgical mJOA scores, is quite wide. The change in mJOA for this sample ranged from -2 to +7 after surgery, with a mean change of +1.88 ± 2.38 (SD). One issue we have identified with using the change in mJOA as a proxy for patient outcome is that the potential for recovery is dependent on initial symptom severity. For example, a patient with an mJOA score of 17 has a much narrower range of outcomes than a patient with an mJOA score of 12 (which could theoretically gain 6 points on the mJOA scale on the way to a complete recovery). This results in the over-weighting of modest recovery in more severe patients – ie. using the example above, if both of these hypothetical patients experienced a change in mJOA of +1 after surgery, the first patient would have experienced a complete recovery, while the second patient would still have severe residual deficits. As such, the change in mJOA is not an ideal measure of patient outcome unless compared between patients with the same initial mJOA score – which would require a substantially larger sample size. However, this is an approach that we are considering for future studies involving larger sample sizes.

Given these limitations, we decided to use the post-surgical mJOA as our measure of patient outcome, since it represents the patient's neurological status in more absolute terms.

Figure 1A: it would be useful to have the number of dropouts and the reasons listed just as you have done for surgery not offered etc.

We thank the reviewer for the suggestion. The number of dropouts and reasons given have now been included in figure 1A.

L316: Do the authors have a reason why there is a decrease between 6 and 12 weeks DCM, as although surprisingly not significant, there is a trend there.

We agree with the reviewer that there does seem to be a downward trend between 6 and 12 weeks in the spinal cord samples. We can only speculate as to why this is the case; however, it is worth noting that this relative decrease in miR-21 expression was not observed in the plasma of these mice, possibly suggesting that this apparent drop in expression may simply be an artifact related to cell death and subsequent changes in cell populations caused by chronic spinal cord compression.

Figure 3A: Did the authors undertake the rotarod on the mice at 2 and 6 weeks of the DCM when the injury were minor and moderate?

We did not perform the rotarod test at 2 and 6 weeks. This was largely due to prior observations using this model, which suggested that the training protocol used to acclimatize the mice to the rotarod acted as a form of physical therapy – and noticeably changed the progression of neurological deficits in the mice. To avoid this potential confounder, we elected to only perform rotarod testing at the experimental endpoint.

Was this a graded effect or was there a significant drop off straight away and then small decreases?

In this DCM model there is typically a graded effect on the locomotor performance. Following recovery from the surgery (usually 1-2 weeks), locomotor deficits are generally not detectable in the wild type

animals. Onset of subtle deficits typically appear from 3-4 weeks, which gradually accumulate until a plateau is reached (>10 weeks).

Figure 3B: I would recommend that the authors change the footprints away from red because of contrast against a black background – alternatively, I would present an inverse on a white background for clarity.

We thank the reviewer for the recommendation and have modified this image based on their recommendation.

Do the authors have any theories about why there are specific changes occurring at 2 weeks generally with the gait analysis but that these differences disappear (aside from stride length)? Is this a real temporary deficit, or is it a case of power of the study?

We believe that this pattern, where miR-21 KO DCM mice develop specific gait abnormalities early (at 2 weeks), is a feature of the mutant phenotype. Part of the explanation for why these gait differences between WT and miR-21 KO DCM do not persist is that the WT DCM mice begin to develop gait abnormalities shortly after 2 weeks, and begin to close the gap. Admittedly, the power of the study may play a role in the timing of this convergence, as some parameters still appear to show smaller, but non-trivial differences at the 6-week time-point. While we cannot necessarily explain the persistence of the differences in stride length between the two genotypes, we believe that this may have something to do with the relationship of stride length to neuropathic pain (Miyagi et al. 2013, Spine. ; Willians Fernando et al. 2020, Neurosci. Lett.). As the miR-21 KO DCM group demonstrated significant hypersensitivity to sensory stimuli in behavioural and electrophysiological testing, this could help to explain some of the more persistent changes in gait, and the contradictory findings between spontaneous locomotion on the catwalk, and "forced" locomotor tasks (rotarod and swimming).

Figure 6: Was this increase in microglia in the DCM and consequently the decrease in the KO mice present at all time points? I am unsure what whether these were on 12 week DCM mice?

Our previous work with this model suggests that the increase in microglia is present at multiple time points as spinal cord compression progresses (Vidal et al, 2017, Karadimas et al, 2013). However, in this study we only counted microglia at the 12-week endpoint. To provide a clearer indication of the timepoint corresponding to the microglial counts in this figure, this information has been added to the figure legend.

While the microglia do not have a different response to pro- or anti-inflammatory cues, are the microglia in the spinal cord preferentially in either of these states? This would tell you also the state of these microglia in the spinal cords through co-labelling. You might be also able to assess this using RT-qPCR on the RNA from the spinal cords, though this would be slightly limited by the mixed cell population in the spinal cord.

In our hands, antibodies against the markers typically used to differentiate between M1 and M2-polarized macrophages (ie. TNFA, CD206, NOS2, ARG1, etc) have been unreliable for in situ characterization of microglial polarization in mouse DCM spinal cords. This matches recent literature, primarily from single cell RNA sequencing, suggesting that the M1/M2 polarization model breaks down in vivo, where microglia/macrophages appear to have far more complex, multidimensional

inflammatory phenotypes (reviewed in RM Ransohoff, Nature Neuroscience, 2016). As such, we did not attempt to characterize microglial polarization beyond isolated microglial culture – where the expression of the aforementioned markers can reliably indicate pro- or anti-inflammatory phenotypes.

While we agree with the reviewer that it could be informative to determine specific differences in the inflammatory phenotype of microglia in the spinal cord, we felt that to do this properly would require the addition of significant resources (single cell RNA-seq) that would ultimately push beyond the scope of this paper.

Reviewer: 2

Comments to the Author
this manuscript covers an interesting topic "the role of MicroRNA-21 in modulation of modulates inflammation and sensorimotor deficits in cervical 2 myelopathy". They found a positive correlation between elevated plasma miR-21 expression, initial symptom severity, and poor treatment outcomes. From animal studies DCM was induced in a miR-21 knockout mouse line. Deletion of miR-21 preserved locomotor function on rotarod and forced swim tests. miR-21 knockout DCM mice also had reduced spinal cord inflammation, demonstrated by the reduction of Iba1+ microglia by approximately 50% relative to wild type DCM controls. However, the paper could be substantially strengthened by addressing the following concern.
In the method section, it is not clear how many mic per group, which age and which sex .

We thank the reviewer for their comments, and have addressed their concerns by including the specific information regarding the number, age, and sex of the mice used in the methods section (pg.7 ln.129-130).

Editorial office comments:
- Please include the sex of mice used in the experiments, whether there are sex effects observed/expected, and if a single sex is used, explicitly state this is a limitation of the study.

The sex of the mice has been included in the "Animals" section of the methods. Since we used only female mice, we have included this as a limitation in the discussion. In addition, we have provided a graph demonstrating the lack of sex differences in miR-21-5p expression in the human DCM samples (Figure S2).

- Figure 3B is very small and the footprints hard to see. Please enlarge and enhance contrast.

We thank the reviewer for this comment. The colour scheme has been inverted (per the request of reviewer 1) and contrast has been increased.

- In fig 3C, please add individual data points for each mouse.

As requested, the individual data points have been added for each mouse.

- in fig 5A, it is difficult to see the images, please provide clearer images.

The size of the images has been increased to improve visibility. Also, the regions of the images containing the swimming mice have been highlighted. Due to the movement of the water, there are some unavoidable light reflection artifacts.

-Please supply a graphical abstract. A graphical abstract is a visual representation of the central finding or methodology of the paper. It will be displayed in the table of contents. Authors should strive to make the graphical abstract informative, interesting, visually appealing, and straightforward (no caption needed, no abbreviations except gene names). Please upload as a separate high resolution image file (tiff preferred). The technical requirements for the graphical abstracts are:

- size of the submitted image: 1200 pixels square at 300dpi
- font of the text: Arial 12-16 point (smaller fonts will not be legible online)

A graphical abstract has been provided.

-Please supply an abbreviated summary of up to 50 words (no abbreviations except gene names). This will be used in the table of contents alongside the graphical abstract. Please upload as a separate word document written in 3rd person, e.g. starting with “First author et al….”

An abbreviated summary has been included with this submission.

- Please check that figures, including those mentioned in the materials and methods section, are numbered in sequential order from the beginning of the article.

All figure references are now sequential in the text. Part of the statistical analysis section has been converted to table in the supplementary information to rectify this issue (Table S3).
