# Supplementary material for: Mir21 modulates inflammation and sensorimotor deficits in cervical myelopathy: data from humans and animal models
Source: Brain Commun. 2021 Jan 21;3(1):fcaa234. doi: 10.1093/braincomms/fcaa234 (PMC7878254; doi:10.1093/braincomms/fcaa234)
Supplement: fcaa234_Supplementary_Data [file Original Submission Decision Letter.pdf › Original Submission Decision Letter.pdf]

Original Submission Decision Letter

26-Oct-2020

Dear Dr. Fehlings,

Thank you for your recent submission BRAINCOM-2020-248 entitled "MicroRNA-21 modulates inflammation and sensorimotor deficits in cervical myelopathy: evidence from humans and animal models" to Brain Communications.

Your manuscript has been reviewed, and while the reviewers and editorial team appreciate many aspects of the paper including the multiple approaches to the problem, they raise some concerns that you will need to address in order for the paper to be considered for publication.

If you can address these concerns, I invite you to submit a revised manuscript and a response to each of the reviewers' comments. The reviewers' comments are included at the bottom of this message.

In addition to the reviewers' comments, please note our editorial comments added below to comply with our requirements for presentation of scientific data at Brain Communications.

Please format your revised manuscript according to our Instructions to Authors https://academic.oup.com/braincomms/pages/General_Instructions – a summary of key points is attached to help with this.

To revise your manuscript, click the link below or log into https://mc.manuscriptcentral.com/braincom and enter your Author Centre, where you will find your manuscript title listed under "Manuscripts with Decisions." Under "Actions," click on "Create a Revision." Your manuscript number has been appended to denote a revision.

*** PLEASE NOTE: This is a two-step process. After clicking on the link, you will be directed to a webpage to confirm. ***

https://mc.manuscriptcentral.com/braincom?URL_MASK=6c44f50f80a0406b9504edc447492a9e

Please upload a revised manuscript with changes tracked with track changes or in coloured text.

When submitting your revised manuscript, you will be asked to respond to the reviewer(s) comments. Please be as specific as possible in your response to all of the comments.

The editorial team at Brain Communications aims to increase transparency in scientific publishing and to demonstrate the value that peer review brings to the scientific process. Please therefore consider whether you are willing to have reviewer(s)' comments on your paper and your response published as supplementary data with the paper if it is accepted. This is entirely optional and will only be done if both the authors and reviewers agree. While submitting your revision there will be an opportunity to review or update the answer you gave previously, if necessary.

IMPORTANT: Your original files are available to you when you upload your revised manuscript. Please delete any redundant files before completing the submission.

Because we are trying to facilitate timely publication of manuscripts submitted to Brain Communications, we would appreciate your revised manuscript as soon as possible. If it is not possible for you to resubmit within 3 months, the option to submit a revision will expire, and we would then consider your revised paper as a new submission. However, if you need a little more time, please get in touch and we can extend this deadline for you.

Once again, thank you for submitting your manuscript to Brain Communications and I look forward to receiving your revision.

Yours sincerely,

Prof. Tara Spires-Jones
Editor, Brain Communications

Reviewer(s)' Comments to Author:

Reviewer: 1

Comments to the Author
Lalibrete and colleagues have investigated the role that miR-21 plays in the progression of degenerative cervical myelopathy through using human plasma samples, a knock-out mouse line, and primary mouse microglia lines. As a whole, the paper provides convincing evidence that miR-21 is a critical target for investigation in DCM. As such, I am pleased to say that I recommend this manuscript to be accepted after addressing the following comments below:

As is standard since 2015, can the authors please put whether they are referring to the -3p or -5p arm of the miRNA throughout the manuscript as doing so in the abbreviations would not be acceptable. Otherwise, I could take this to mean that the precursor is being referred to and that both -3p and -5p are involved.

Introduction:
Are there any other miRNA associated with DCM? You only mention miR-21 and I am wondering whether there are others that have been linked previously, and if so, why these were investigated during the human plasma study. If not though, it would still be useful to have this stated in the introduction.

Methods:
L161: What was the volume of plasma that was utilised in extracting the samples? Even if it was the default amount stated in the instructions, it is good to state these for clarity especially as the starting volume can be up to 900ul.
L165: Consequently, how much RNA was loaded into the cDNA reactons? In terms of serum, was this the recommended starting volume amount?
L171: There is no such thing as real-time quantitative PCR – real time and quantitative describe the same thing – RT should be referring to the fact you did a reverse transcription step.
L176: How were your normalisers chosen? Normalisers are extremely difficult to select for in plasma data without having undertaken screening experiments like RNA-seq as miRNA can be highly variable between samples. Can you please demonstrate that they were not changed between your samples and were stable across your samples through providing average ± SD of the raw Cq values.
L177: For all mRNA TaqMan probes and primer sets, please provide the catalog numbers as there are multiple primer sets for each gene, so these must be provided at least in a supplementary table.
L202: For the Catwalk Gait analysis, how many runs were not analysed because they were interrupted? I am curious whether in addition to their disrupted gait whether they also had increased stop/start movements.

Results:
L300: I am not sure that a correlation between post-surgical mJOA and miR-21 dCt show anything. If they already have a low score, how much can this change post-surgery? I think a more useful analysis would be to look at the change in score before and after surgery and compare, as if there is an correlation between pre-surgery miR-21 dCt and post-surgery mJOA, then it should show a correlation.
Figure 1A: it would be useful to have the number of dropouts and the reasons listed just as you have done for surgery not offered etc.
L316: Do the authors have a reason why there is a decrease between 6 and 12 weeks DCM, as although surprisingly not significant, there is a trend there.
Figure 3A: Did the authors undertake the rotarod on the mice at 2 and 6 weeks of the DCM when the injury were minor and moderate? Was this a graded effect or was there a significant drop off straight away and then small decreases?
Figure 3B: I would recommend that the authors change the footprints away from red because of contrast against a black background – alternatively, I would present an inverse on a white background for clarity.
Do the authors have any theories about why there are specific changes occurring at 2 weeks generally with the gait analysis but that these differences disappear (aside from stride length)? Is this a real temporary deficit, or is it a case of power of the study?
Figure 6: Was this increase in microglia in the DCM and consequently the decrease in the KO mice present at all time points? I am unsure what whether these were on 12 week DCM mice?
While the microglia do not have a different response to pro- or anti-inflammatory cues, are the microglia in the spinal cord preferentially in either of these states? This would tell you also the state of these microglia in the spinal cords through co-labelling. You might be also able to assess this using RT-qPCR on the RNA from the spinal cords, though this would be slightly limited by the mixed cell population in the spinal cord.

Reviewer: 2 (Maryam Ardalam)

Comments to the Author
this manuscript covers an interesting topic “the role of MicroRNA-21 in modulation of modulates inflammation and sensorimotor deficits in cervical 2 myelopathy”. They found a positive correlation between elevated plasma miR-21 expression, initial symptom severity, and poor treatment outcomes.

From animal studies DCM was induced in a miR-21 knockout mouse line. Deletion of miR-21 preserved locomotor function on rotarod and forced swim tests. miR-21 knockout DCM mice also had reduced spinal cord inflammation, demonstrated by the reduction of Iba1+ microglia by approximately 50% relative to wild type DCM controls. However, the paper could be substantially strengthened by addressing the following concern.
In the method section, it is not clear how many mic per group, which age and which sex .

Editorial office comments:
- Please include the sex of mice used in the experiments, whether there are sex effects observed/expected, and if a single sex is used, explicitly state this is a limitation of the study.
- Figure 3B is very small and the footprints hard to see. Please enlarge and enhance contrast.
- In fig 3C, please add individual data points for each mouse.
- in fig 5A, it is difficult to see the images, please provide clearer images.
-Please supply a graphical abstract. A graphical abstract is a visual representation of the central finding or methodology of the paper. It will be displayed in the table of contents. Authors should strive to make the graphical abstract informative, interesting, visually appealing, and straightforward (no caption needed, no abbreviations except gene names). Please upload as a separate high resolution image file (tiff preferred). The technical requirements for the graphical abstracts are:
- size of the submitted image: 1200 pixels square at 300dpi
- font of the text: Arial 12-16 point (smaller fonts will not be legible online)

-Please supply an abbreviated summary of up to 50 words (no abbreviations except gene names). This will be used in the table of contents alongside the graphical abstract. Please upload as a separate word document written in 3rd person, e.g. starting with “First author et al….”
- Please check that figures, including those mentioned in the materials and methods sectiion, are numbered in sequential order from the beginning of the article.
