# Supplementary material for: Mir21 modulates inflammation and sensorimotor deficits in cervical myelopathy: data from humans and animal models
Source: Brain Commun. 2021 Jan 21;3(1):fcaa234. doi: 10.1093/braincomms/fcaa234 (PMC7878254; doi:10.1093/braincomms/fcaa234)
Supplement: fcaa234_Supplementary_Data [file Revision 1 Decision Letter.pdf › Revision 1 Decision Letter.pdf]

Revision 1 Decision Letter

27-Nov-2020

Dear Dr. Fehlings,

It is a pleasure to accept your manuscript entitled "MicroRNA-21 modulates inflammation and sensorimotor deficits in cervical myelopathy: data from humans and animal models" for publication in Brain Communications. Any comments made by the reviewer(s) are included below.

The next step is for your paper to go through "First Look". We will contact you again shortly if any final files or formatting changes are required for publication or if we have any queries.

If no changes are needed, your submission will be transferred to our Production Department and you will hear from the Production Editor in due course.

Video abstracts - Brain Communications encourages authors to provide a video abstract to accompany their article. These are hosted on the Brain/Brain Communications YouTube channel. If you are interested in producing a 5-min video abstract to promote your article, then please contact the Editorial Office (brcoms.editorialoffice@oup.com) for further details and guidelines.

Thank you very much for your contribution. On behalf of the Editors of Brain Communications, we look forward to your future contributions to the Journal.

Yours sincerely,

Professor Tara Spires-Jones
Founding Editor, Brain Communications

Reviewer(s)' Comments to Author:

Reviewer: 1

Comments to the Author
I thank the authors for taking the time to go through my comments and address each of them. While some of the explanations that are provided in the comments such as the discussion about the downward trend in miR-21 expression and the specific changes at 2 weeks with gait analysis would be useful to have in the paper itself to provide clarity to readers, I feel the comments have been well addressed. Therefore, I am happy to recommend that this paper be accepted for publication.
